# Tracking the phage trends: A comprehensive review of applications in therapy and food production

**DOI:** 10.3389/fmicb.2022.993990

**Published:** 2022-11-24

**Authors:** Anu Bala Jaglan, Taruna Anand, Ravikant Verma, Medhavi Vashisth, Nitin Virmani, B. C. Bera, R. K. Vaid, B. N. Tripathi

**Affiliations:** ^1^Department of Zoology and Aquaculture, Chaudhary Charan Singh Haryana Agricultural University, Hisar, India; ^2^ICAR – National Research Centre on Equines, Hisar, India; ^3^Department of Molecular Biology, Biotechnology, and Bioinformatics, Chaudhary Charan Singh Haryana Agricultural University, Hisar, India; ^4^Animal Science Division, Indian Council of Agricultural Research, Krishi Bhawan, New Delhi, India

**Keywords:** antibiotic resistance, bacteriophage, compassionate phage therapy, biocontrol, aquaculture

## Abstract

In the present scenario, the challenge of emerging antimicrobial resistance is affecting human health globally. The increasing incidences of multidrug-resistant infections have become harder to treat, causing high morbidity, and mortality, and are posing extensive financial loss. Limited discovery of new antibiotic molecules has further complicated the situation and has forced researchers to think and explore alternatives to antibiotics. This has led to the resurgence of the bacteriophages as an effective alternative as they have a proven history in the Eastern world where lytic bacteriophages have been used since their first implementation over a century ago. To help researchers and clinicians towards strengthening bacteriophages as a more effective, safe, and economical therapeutic alternative, the present review provides an elaborate narrative about the important aspects of bacteriophages. It abridges the prerequisite essential requirements of phage therapy, the role of phage biobank, and the details of immune responses reported while using bacteriophages in the clinical trials/compassionate grounds by examining the up-to-date case reports and their effects on the human gut microbiome. This review also discusses the potential of bacteriophages as a biocontrol agent against food-borne diseases in the food industry and aquaculture, in addition to clinical therapy. It finishes with a discussion of the major challenges, as well as phage therapy and phage-mediated biocontrols future prospects.

## Introduction

**Figure 1:**
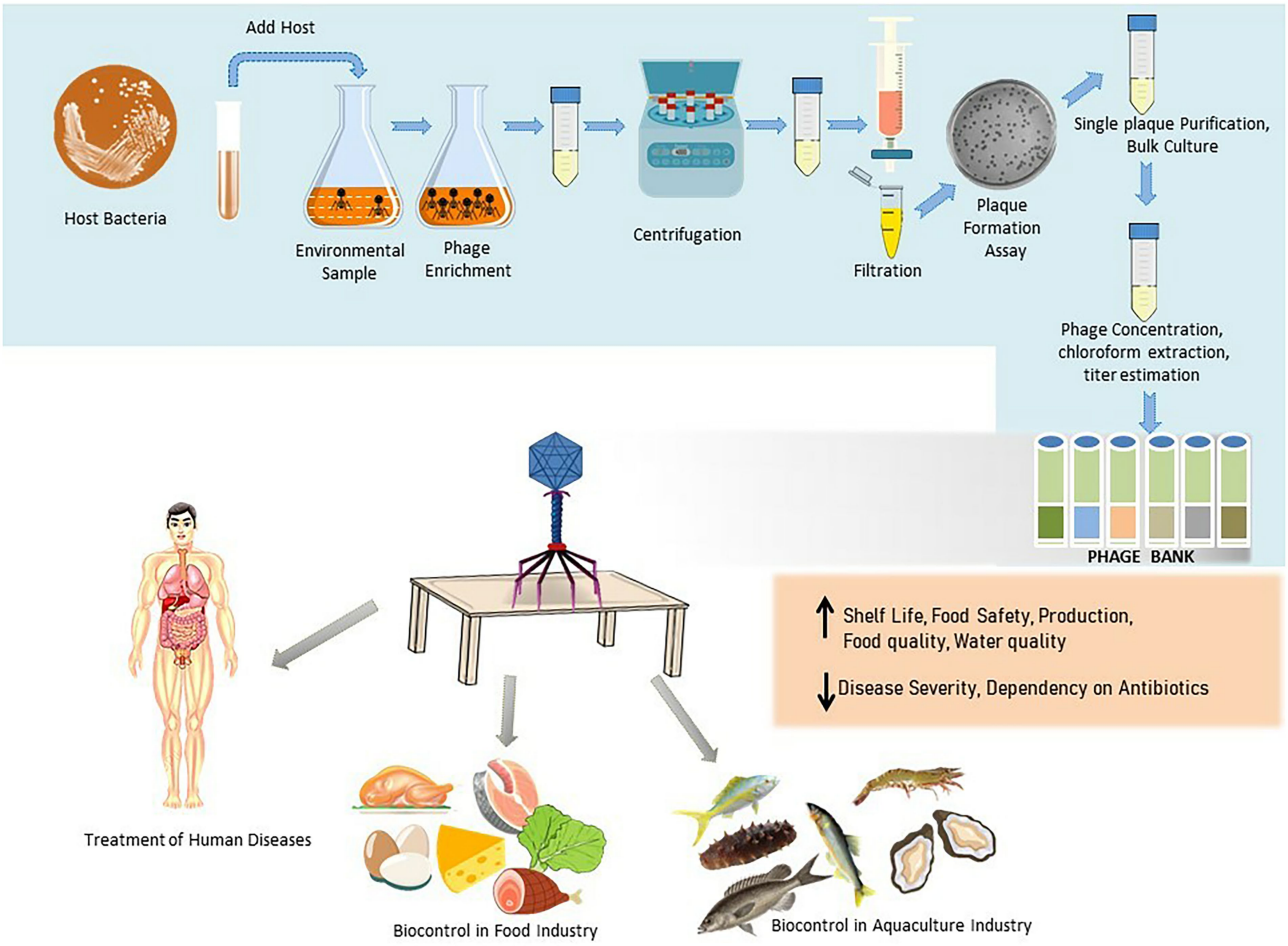
Schematic illustration of the phage isolation, purification, preservation, and their applications in therapy and biocontrol in food industry and aquaculture.

Globally, throughout the ages, the primary factor that leads to mortality is infectious diseases. Although infectious disease incidences have been reduced with the advancement in sanitary and hygienic conditions, diseases continued to be a significant threat until the discovery of antibiotics in 1928. The commercialization of antibiotics is regarded as a medical miracle leading to a rise in average life expectancy up to 78.8 years in the United States (National Center for Health Statistics and Center For Disease Control And Prevention, 2017), and in England mortality due to infectious disease shrank from 25 to 1% (Smith et al., 2012). During the “golden age” of antibiotics, from 1940 to 1960, most of the antibiotic classes that are used today were discovered, and it was speculated that infectious diseases would soon be under control. In fact, in the year 1970, an epidemiological transition to shift national resources from infectious diseases to treating chronic diseases was observed (McCallum and Mathers, 2017). Nevertheless, along with the increased use of antibiotics, the bacterial pathogens became increasingly resistant to the commonly used antibiotics, stressing the researchers to fight the battle, which seemingly escalated in favor of the bacteria (Alumran et al., 2011; Wang et al., 2019). These concerns provoked the WHO in the year 2015 to launch a Global Action Plan on antimicrobial resistance (AMR). AMR is a significant cause of morbidity, and mortality in the Asia Pacific region, home to more than half of the worlds population (Yam et al., 2019). Each year antibiotic-resistant bacterial and fungal infections leave 2.8 million people infected, causing the death of 35,000 in the United States alone (Centers for Disease Control and Prevention (U.S.), 2019). In India, nearly 58,000 neonatal deaths occur due to infections caused by microorganisms resistant to first-line antibiotics (Laxminarayan et al., 2016). Unfortunately, improper management of antimicrobial agents has led to unexpected complications due to resistant microbes that we face today. The increasing incidence of bacterial antimicrobial resistance affecting human and animals are related to the consumption of irrelevant antimicrobial drugs, self-medication, and over the counter availability of antibiotics. In food animals, antibiotics have been used for several years to treat and prevent microbial infections and as growth promoters. Several studies (Marshall and Levy, 2011; Founou et al., 2016; Ma et al., 2021) reported that resistant bacteria were transferred from food animals to humans. Thus, in the search for alternative strategies to control bacterial infections, revisiting the practice of phage therapy became one of the more popular suggestions. The advantages that phages offer over antibiotics in the therapy and biocontrol of pathogens in the food industry and aquacultures (Figure 1) are due to their characteristics such as host specificity, self-amplification, natural origin, easy isolation, low toxicity, and the potential to degrade biofilms (Topka et al., 2019).

## Status of phage therapy

Bacteriophages are believably one of the most ancient and ubiquitously existing biological entities (viruses) that are capable of infecting and replicating within bacteria and therefore play an important role in sustaining the equilibrium of an ecosystem where bacteria reside. After injecting their DNA, phages use the host machinery to replicate and translate the necessary information required to produce viral progeny (in the case of lytic phages) and enzymes such as spannin, holin, and endolysin. In the case of gram-negative hosts, caudovirales use holin-endolysin or pinholin SAR (Single arrest release) mechanisms for breaking the peptidoglycan bond of the cell wall, resulting in host lysis and the release of viral progeny during lytic cycle (Abdelrahman et al., 2021). While in lysogenic/temperate phages, the viral genome integrates into the host genome and multiplies with them for many generations.

The discovery of bacteriophages is independently attributed to F.W. Twort and felix dherelle. Soon after their discovery, phages were used for the control of infectious diseases in the pre-antibiotic era. However, the discovery of antibiotics ceased the therapeutic use of bacteriophages in the Western world. In recent years, the failure of conventional antibiotics in controlling and treating the pathogenic strains of the bacteria, and the paucity of other therapeutic preferences has hastened the resurgence of bacteriophage therapy. Phage therapy seems to be more effective when it is employed for targeting bacterial infections in the early stages when bacteria have not entered the phage-resistant physiological states (Abedon, 2016). However, in clinical practice, phage therapy has been used for the successful treatment of severe or incessant bacterial infections (Abedon, 2019). Successful treatment of chronic infections with phage therapy has been majorly reported in Georgia, Poland, and Russia. The success of the therapy depends upon the type of infection, type of phage or phage-based products, phage dose, and route of administration. Phage therapy has been carried out as a last resort in the cases where antibiotic therapy failed (as shown in Supplementary Table S1) and has not been validated yet by clinical trials. In spite of the widespread pervasiveness, specificity, and activity against antimicrobial-resistant and biofilm-forming bacteria, phage therapy is not weighed as a mainstream treatment but just a complementary approach (Międzybrodzki et al., 2012; Doub et al., 2020). There are no specific universal regulations for the application of phage therapy in treatment regimens. Regulatory authorities throughout the globe have adopted different guidelines for using phage therapy in clinical cases. In the United States patients are treated under emergency investigational new drug (eIND) approval from FDA, in Australia specific access scheme is adopted, and in Belgium, a magistral phage approach is being followed (Donovan, 2017; Jarow et al., 2017; Pirnay et al., 2018; Djebara et al., 2019). These non-uniform regulations among different countries have constrained the adoption of phage therapy in general population. However, recently in the United States, the first phage therapy center, UC San Diego Center for Innovative Phage Application and Therapeutics has been established and several patients were successfully treated with phage therapy and no side effects were observed (Aslam et al., 2020). Similarly, several clinical trials were carried out in European countries and results show that phage therapy is well tolerated with no or mild adverse effects. In addition, several clinical trials are being recruited for the treatment of patients suffering from urinary tract infections complicated by *E.coli* and *K. pneumoniae*, cystic fibrosis with chronic *Pseudomonas aeruginosa* infections, and prosthetic joint and osteomyelitis infections due to *Staphylococcus aureus* (Clinicaltrials.gov). The Shanghai Institute of Phages in China has also started providing clinical treatment with phages to patients suffering from multidrug-resistant infections (Wu et al., 2021).

Along with the whole phages, phage-derived lysins and genetically modified phage (GMO phage)/lysins are used in the therapy and biocontrol. GMO phages are considered Advanced Therapy Medicinal Product (ATMP) by the European Medicines Agency. For their access to the European market, they must comply with Directive 2001/83/EC, Directive 2001/18/EC (Article 12.2), and Regulation (EC) 726/2004 (Articles 6.2 and 6.3) under current good manufacturing practices (cGMP) (European Commission, 2019). However, in the European Union, the implementation of GMO regulation varies from state to state, resulting in uncertainties in accessing the market in some states. While in the United States, GMO phages are subject to 21CFR312, under the Office of Tissues and Advanced Therapies (OTAT), and natural phages are supervised by the Office of Vaccines Research and Review (OVRR) (Food and Drug Administration, 2019). The Food and Drug Administrations (FDA) Centre for Food Safety and Applied Nutrition (CFSAN) provided Generally Recognized as Safe (GRAS) status to the approved phage or phage-based product for application in the food industry. Similarly, in several other countries such as Australia, Canada, Israel, New Zealand, and Switzerland, phage formulations get approval based on US regulations for food safety purposes. Through the International Conference on Harmonization (ICH), European, American, Japanese, and other regulatory bodies obtained common guidelines to aid in the development of pharmaceuticals.

## The essential requirements for phage therapy

Several studies have indicated the successful treatment of bacterial infections with phage therapy (Doub et al., 2020; Cano et al., 2021; Johri et al., 2021). However, others have reported the modest effectiveness of phage therapy (Rose et al., 2014; Ooi et al., 2019). As such, there is a need to establish a standard operating procedure that will ensure the success of phage therapy. Therefore, essential prerequisites for successful outcome of phage therapy are divided based on clinical and laboratory indications. The exclusion of the nonbacterial infection cases, as well as the sequestration and identification of infection-causing agents, are clinical prerequisites. The laboratory prerequisites include the identification by culture-based or non-culture-based methods like MALDI-TOF, PCR, gene sequencing, immunological methods, and screening of the sensitivity of the bacterial isolate to phages available in the phage biobank or isolated from environmental sources for application in therapy.

### Phage isolation and purification

The main rule for finding a bacteriophage for a specific host is to use an environmental sample where the host is located. For example, phages for fish pathogens are generally isolated from coastal or fish farm waters (Yu et al., 2013). Similarly, phages for mammalian intestinal bacteria are isolated from fecal matter and sewage. The primary method for phage isolation is the enrichment procedure, which remains unchanged since it was adopted by Felix d’Herelle. However, some researchers use direct plating of environmental samples with the bacteria on the agar plate and check for plaque formation, which is then purified (Gencay et al., 2017). But for the success of this method, it is required that the samples have a high concentration of phage. Therefore, plating after concentration or enrichment is generally the method of choice. Different morphology and size of plaques in the double-layered agar plate indicate the presence of more than one phage type in the sample (Hyman, 2019). Apparently, different single plaques are purified individually against their respective host bacterium. Lastly, a scale-up using bulk culture is used to prepare a phage library that is specific to the host bacteria (García et al., 2019). This library is then stored until it is needed.

### Characterization of bacteriophages

Phage characterization generally includes the determination of the plaque morphology, host range and the multiplicity of infection. Structural properties of phages are determined by transmission electron microscopy, thermal and pH stability for therapeutic purpose, and genome/proteome analysis are carried out for checking their suitability for therapy. A plaque assay is performed to assess the lytic efficiency of a bacteriophage where phages producing clear plaques are generally considered lytic/virulent while those forming turbid plaques are considered lysogenic/temperate. Phages can be additionally screened for the presence of integration/recombination/excision/toxin genes that are associated with the temperate life style and have the potential for transduction, which can be tested either by nucleic acid hybridization/PCR or by whole phage genome sequencing (Russell, 2018). Whole-genome sequencing can also help describe the enzymes such as depolymerase, virion-associated lysin, and endolysins that are encoded in their genome which exhibit therapeutic potential (Roach and Donovan, 2015) and be expressed and purified for use in therapy and biocontrol.

### Purification and preparation of bacteriophage for therapy

Recently, the demand of phages (or phage-based products) for their use in human, veterinary, aquaculture, and processed foods has increased, but only a limited number of phage products are developed according to guidelines under Good Manufacturing Practices (GMP). To prepare a phage for its use in therapy, it needs to be free of bacterial endotoxins and other gross impurities while concurrently maintaining the phage efficacy. Endotoxins and protein toxins are the main impurities present in phage lysate that need to be removed from therapeutic preparations. Chromogenic Limulus Amebocyte Lysate (LAL) assay (Abate et al., 2017) is used to quantify the level of endotoxins in a phage preparation. For the intravenous application of any pharmaceutical or biological product, FDA has limited the level of endotoxin to be less than 5 EUs·kg^−1^ h^−1^ (Food and Drug Administration, 2018). The traditional methods to purify phages involving the use of cesium chloride (CsCl) based density gradient ultracentrifugation have been reported to increase the LPS quantity in phage preparations by 30% (Luong et al., 2020). Therefore, the purification methods like dead-end filtration, cross-flow filtration, extraction with 1-octanol (Bonilla et al., 2016), EndoTrap HD column, Pierce column, ion exchange (Adriaenssens et al., 2012), and LPS-affinity chromatography, are being used to successfully remove the endotoxins while maintaining high phage titer (Luong et al., 2020). Depending on the type and severity of the disease and route of phage administration, different formulation techniques are adopted for targeted delivery and successful release of phage at the site of infection. Phage formulation for topical application includes liposomal encapsulation, attachment with electrospun nanofibres, or as gel-, cream-based emulsions. For oral delivery, phages may be encapsulated in polymers like alginate, eudragit, chitosan, and used as spray-dried powders (Vinner and Malik, 2018). For intranasal route of phage delivery, aerosols preparation using nebulization has been used (Chang et al., 2018). For the intravenous route of administration, phages may be delivered through injection and infusion. Quality control (QC) for the release of phage products includes assessment of phage identity, purity, and safety for checking that the final product is free of any impurities, followed by testing phage viability to check the loss of titer during product preparation and formulation (Moraes de Souza et al., 2021).

### Phage susceptibility testing

A phage susceptibility test is carried out to simultaneously check hundreds of phage candidates selected from different phage banks and phage laboratories against the bacteria isolated from a patient. A phage susceptibility test can be performed by conventional agar overlay assay, or by direct spot of liquid phage suspension on the targeted bacterial lawn (Direct spot assay). Further, the productivity of phage infection can be evaluated by the Efficiency of Plating (EOP) assay, by studying time kill growth kinetics in for planktonic cultures and the biofilm inhibition/eradication assay (Haines et al., 2021). A phage susceptibility test will identify one or more phages for cocktail design or synergy assessment which may be carried forward as potential candidates to treat the infection.

### Storage condition

For a successful phage therapy, an essential requirement is the phage stability, which differs with different kinds of bacteriophages. Any excellent long-term preservation method has not been described yet for phages. In several cases, there has been no significant decrease in phage titer after a few days of preservation and titers could be maintained for years (Weber-Dąbrowska et al., 2016). During storage, temperature, humidity, and access to light should however be controlled according to individual phage properties. As the phage coat consists of protein, which gets denatured at high temperatures, leading to the biological inactivation, a phage that remains stable under storage conditions is a suitable candidate for bacteriophage therapy. In the lysate form, phages generally maintain their activity when preserved at room temperature, 4°C (Merabishvili et al., 2009; Weber–Dąbrowska et al., 2016), −20°C, and at −80°C with glycerol or in liquid nitrogen. However, ice crystal formation at −20°C reduces the viability of the phages, which can be improved with the inclusion of glycerol. Tovkach et al. (2012) defined the STMG buffer composition for the sensitive phages, providing long-term stability for storage at 4°C to −2°C. To preserve *Caudovirales*, Golec et al. (2011) provided a safe method of storage, i.e., phage-infected bacterial cells with no significant loss of phage activity. The authors proposed it as a suitable method for the phages where structure, biology, and multiplication requirements have not been met yet. Several studies have supported this idea of phage preservation (Łobocka et al., 2014; González-Menéndez et al., 2018). Regarding, lyophilization, Puapermpoonsiri et al. (2010) observed a correlation between the lytic activity of phages and moisture content in lyophilized powder. The authors observed that phage encapsulation based on emulsification and freeze-drying leads to only a partial loss of phage lytic activity due to their exposure to the water-dichloromethane interface and not due to lyophilisation process itself. The losses that occurred due to lyophilization have been overcome by the addition of albumin, gelatin, or salts that act as a stabilizer (Alfadhel et al., 2011). The addition of, for example, yeast extract, lecithin, and raw egg white during the lyophilization of sensitive phages has been shown to increase their stability, and the dried powder obtained after lyophilization remained stable during 12 months of storage at 37°C (Schade and Caroline, 1943). Similarly, stabilizers like sucrose, trehalose, mannitol, glycine, polyvinylpyrrolidone, and PEG 6000 (polyethylene glycol) at different concentrations were used for the lyophilization of phages against *S. aureus*, including MRSA strains and it was found that trehalose and sucrose (0.5 M) were the best additives for protecting the phage particles (Merabishvili et al., 2013). In addition, the encapsulation of the bacteriophage increases the stability and improves the targeted delivery of the bacteriophages for the therapeutic applications (Loh et al., 2021).

## Role of phage biobank

**Figure 2:**
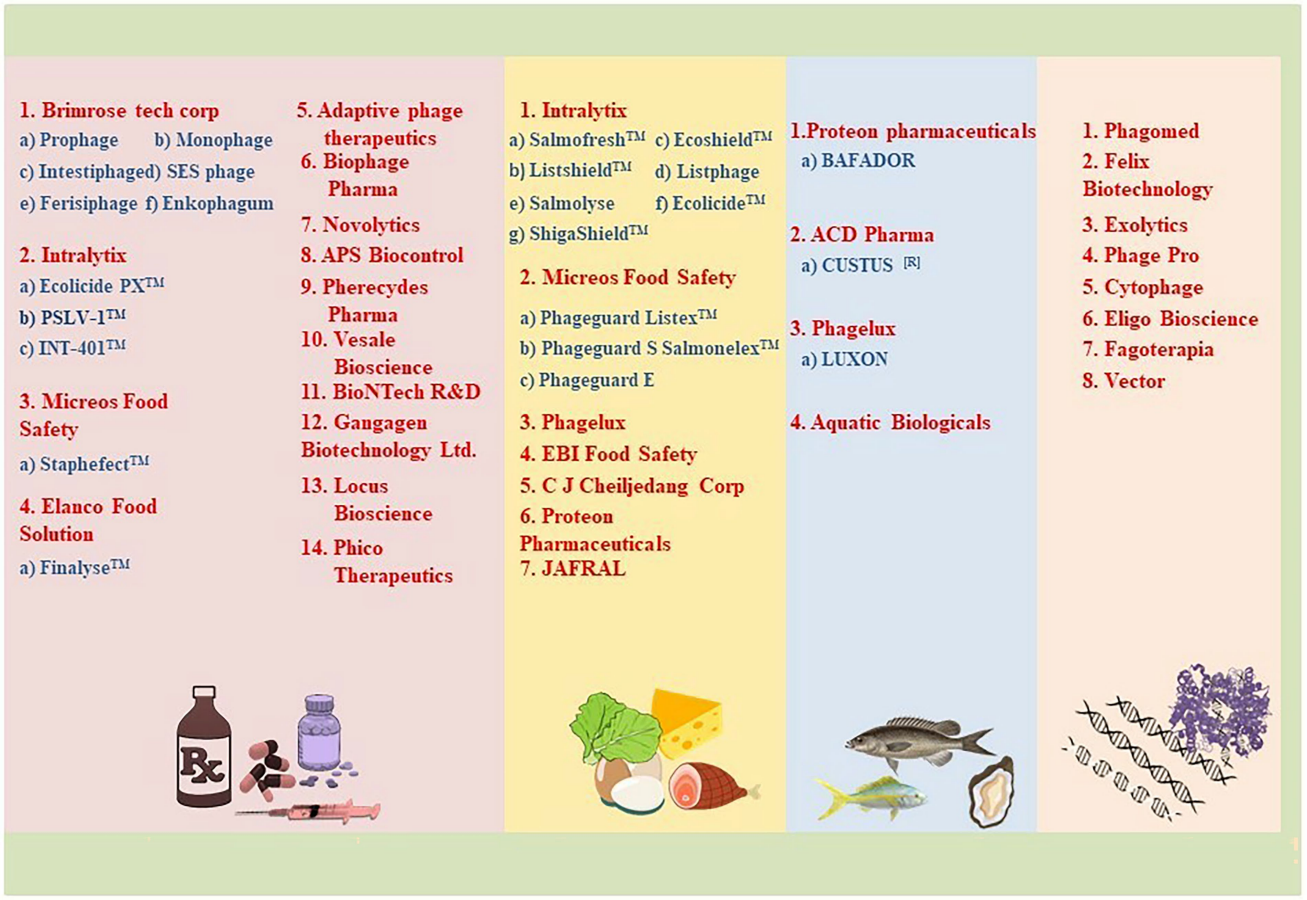
Illustration of companies involved in the production of natural and synthetic phage/phage-based products in various sectors.

Phage biobanks contain a variety of phages that are well characterized and are ready for use in compassionate and clinical trials (Yerushalmy et al., 2020). The best known are the Eliava Institute of Bacteriophages, Microbiology, and Virology in Georgia (with a collection of ˃1,000 phages), and the Hirszfeld Institute of Immunology and Experimental Therapy (˃850 phages in the collection) in Poland where over the counter phage cocktails for the generalized bacterial disease are provided to patients (Żaczek et al., 2020). In addition, in the recent few years, Government grants and public funding helped in the establishment of more phage biobanks for therapy in both compassionate and clinical cases. The lists include Baylor College of Medicine, Navy Medical Research Centre, and the Walter Reed Army Institute of Research in the U.S., Queen Astrid Military Hospital in Belgium, Fagenbank in Netherlands, Hebrew University and Hadassah Medical Centre in Israel, and the Australian Phage Biobank. Apart from these, several phage banks are established for laboratory research purposes, details of which are provided in Table 1. Several companies, as shown in Figure 2, are also actively involved in the collection and formulation of phage-based product sand taking efforts for making them commercially available for therapy. For the application of phages in clinical use, a strict enrolment criterion issued by the International Organization of Standardization (ISO), the Minimum Information About Biobank Data Sharing (MIABIS), and the International Society for Biological and Environmental Repositories (ISBER) needs to be assessed and only those phages that meet these criteria should be used in the therapy to avoid any potential safety risks. The Phage biobanks may play an important role in phage therapy by timely presenting a panel of characterized phages against emerging multidrug-resistant bacteria. But for a successful phage therapy, a roadmap to link phage biobank with pathogen biobank, and building capacity for surveillance programs by sharing protocols, research, and materials is required (Lin et al., 2021). Along with this, there is a need to address the ethical and legal hassles. The initiative of phage directory has enabled communications related to the search for therapeutic phages amongst other repositories globally and has provided a platform for the biobanks to interact.

**Table 1:** Status of phage collections worldwide.

Phage collection name	Country	Number of phages	Host	Link	Year of establishment
Adaptive phage therapeutics (APT)	United States	_˜_1000	Mainly the ESKAPE pathogens	http://www.aphage.com/the-science/	2010
The felix d’Herelle reference center for bacterial viruses	Canada	˃400	*Bacillus, Escherichia, Lactococcus, Pseudomonas, Salmonella, Sinorhizobium, Staphylococcus* spp.	http://www.phage.ulaval.ca/en/phages-catalog/	1982
The bacteriophage bank of Korea	South Korea	1964	*E. coli*, *P. aeruginosa*, *Salmonella* spp., *Staphylococcus aureus*, *Listeria monocytogenes*, *Acinetobacter baumannii*, *Enterococcus* spp. etc.	http://www.phagebank.or.kr/intro/eng_intro.jsp	2010
Leibniz Institute-DSMZ (German collection of microorganisms and cell culture)	Germany	1,000	*Escherichia* spp., *Bacillus* spp., *Pseudomonas* spp., *Serratia* spp., *Salmonell*a spp. etc.	https://www.dsmz.de/collection/collection-experts	1969
ATCC bacteriophage collection	United States	350	A few dozen hosts	https://www.atcc.org/microbe-products/virology/bacteriophages#t=productTab&numberOfResults=24=[Bacteriophage]	1925
NCTC bacteriophage collection	United Kingdom	97	*Streptococcus agalactiae, Staphylococcus aureus, E. coli* and*Campylobacter*	https://www.phe-culturecollections.org.uk/products/bacteria/bacteriophages.aspx	2019
Actinobacter phage database	United States	20,669	Species from Actinobacteria phylum mainly including genus *Mycobacterium*, *Microbacterium*, *Gordonia*, *Arthrobacter*	https://phagesdb.org/data/	2010
Fagenbank, TU Delft	Netherlands	120	Unknown	https://www.tudelft.nl/en/delft-university-fund/	2019
AHU culture collection	Japan	35	Mainly against *Vibrio* spp., *Roseobacter* spp., *Bacillus* spp.	http://ahu1.agr.hokudai.ac.jp/index.html	ND
Israeli Phage Bank (IPB)	Israel	˃300	16 different species mainly including *P. aeruginosa*, *S. aureus*, *Propionibactrium acnes* etc.	https://ronenhazanlab.wixsite.com/hazanlab/the-404-israeli-phage-bank	ND-
Monash Phage Foundry (MPF)	Australia	Unknown	Unknown	https://research.monash.edu/en/projects/the-monash-phage-foundry	2021
Millard lab, University of Leicester	United Kingdom	˃9000	Unknown	http://millardlab.org/bioinformatics/bacteriophage-genomes/	ND

## Clinical trials of phage therapy

In Eastern Europe and the former Soviet Union, phages are widespread and integrated with the health care systems. However, meticulous scientific standards must be used to select and integrate phages in clinical trials. For a successful phage therapy, it is important to decipher the etiology of the disease (i.e., whether mono or polymicrobial) (Villarroel et al., 2017) and accordingly, selecting and using the already well characterized phages or isolating a potentially lytic phage. The process of identifying bacterial species causing infection and selecting a specific phage against it, requires research and financial efforts in a typical hospital setting, compared to antibiotics that can be prescribed immediately (Schmidt, 2019). Additional information such as formulations, dosing, and efficacy of phages also need to be assessed before their use in the clinical trials (Payne and Jansen, 2003; Harper, 2018). Moreover, phage therapy in humans faces many challenges due to lack of legalized and competently disciplined clinical trials. Planning and designing the clinical trials with phages, becomes more complicated in the absence of the regulations specific for phage therapy. Dose dependent pharmacokinetics of bacteriophages, according to the exact standards of drug based clinical trials, is difficult to monitor due to their self-replicating nature which is not the case with chemical antibiotics. Some of the researchers propose the argument that as human exposure to bacteriophages occurs every day, and they help shape the human gut microbiome hence they appear safe as a therapeutic option in comparison to antibiotics (Manrique et al., 2016). However, many issues need to be addressed as the supportive background of before clinical trials with phage. The first is about excluding toxins and bacterial debris from phage preparations according to the quality parameters (Parracho et al., 2012). Secondly, the bactericidal effect of phages leads to the onset of toxic shock (Furfaro et al., 2018), and as such, lytic efficiency matters. Several studies witnessed the clinical trials of phage therapy attempted in Georgia, Poland, and Australia for treating burn wounds, chronic otitis, diarrhea, urinary tract infections, venous leg ulcer, and bacteremia, addressing the safety and efficacy of phages in humans (Kutter et al., 2010; Petrovic Fabijan et al., 2020). In 2005, 15 healthy volunteers received *E. coli* phage T4 without any adverse events, which was the first clinical trial as per the modern English literature (Bruttin and Brüssow, 2005). Later the evaluation of the safety of phage therapy in bacterial infections was carried out in several other clinical trials. Among these, a randomized, placebo-controlled clinical trial was conducted with 113 patients, out of which 97 patients divided into three groups (28 pyophage, 32 placebo, and 37 antibiotics) received their allocated treatment for 7 days. Success rates of the treatment did not differ significantly as 5 patients in pyophage group, 9 in placebo group, and 13 in the antibiotic group showed normalization of urine culture, while adverse events were observed in 6 patients of the pyophage group compared with 13 in the placebo group and 11 in the antibiotic group (Leitner et al., 2021). Bangladeshi children hospitalized with acute bacterial diarrhea received T4 coliphage or a commercial Russian coliphage product or placebo orally for 4 days and showed no improvement in diarrhea outcome however no adverse events were observed (Sarker et al., 2016). Similarly, a nine-coliphage cocktail at two different concentrations and a placebo were given orally to 15 healthy volunteers and no adverse events were observed in the liver and kidney functioning (Sarker et al., 2012). A similar randomized, double-blind, placebo-controlled clinical trial with 43 healthy adults suffering from self-reported gastrointestinal distress, was conducted by treating them with one 15-mg capsule containing a cocktail of four bacteriophages for 28 days and significant improvement in the symptoms of gastrointestinal distress and a decrease in the level of aspartate aminotransferase were observed in this case (Gindin et al., 2018). Through all these studies and some others (Sarker et al., 2017; Febvre et al., 2019; Grubb et al., 2020) it was demonstrated that bacteriophage reduces the harmful bacteria without disrupting the normal gut microbiota and improves the overall gut health. The patients suffering from chronic rhinosinusitis were divided into 3 cohorts (3 patients/cohort) and the patients received intranasal irrigation with different AB-SA01 concentrations for 7–14 days. The results showed that intranasal bacteriophage treatment was well tolerated with no adverse events except for a small decrease in bicarbonate level in a patient. Complete eradication of bacterial infections was observed in two out of the nine patients (Ooi et al., 2019). Similarly, several other studies reported the safety or the efficacy of phage therapy in the eradication of the *Pseudomonas aeruginosa, Staphylococcus aureus, Enterococcus, E.coli,* and *Clostridium defficile* infections (Rhoads et al., 2009; Wright et al., 2009; McCallin et al., 2013; Leitner et al., 2017; Zuo et al., 2018; Jault et al., 2019; Petrovic Fabijan et al., 2020). Also, the phase I/II/III clinical trials with the phage endolysins in the adult participants showed that phage endolysin-based drugs are well tolerated with mild adverse events such as headache, fatigue, rigors, and myalgiain a few participants (Danis-Wlodarczyk et al., 2021; Wire et al., 2022). Detailed information on all the registered clinical trials of phage therapy is available online at https://globalclinicaltrialdata.com and https://clinicaltrials.gov.

## Compassionate phage therapy

Compassionate therapy refers to using non-standard medicines to treat a patient for which authorized medicines have run out. Article 37 of the “Helsinki Declaration” summarizes the doctrine of the compassionate use of phage therapy and emphasizes the physicians consent to use best practice to treat the patient along with the patients or guardians consent (World Medical Association, 2013). The regulatory authorities such as FDA in the United States, the Therapeutic Goods Administration (TGA) in Australia, and French National Agency for Medicines and Health Products Safety (ANSM) in France have approved the cPT under eIND, special access schemes, or temporary use authorization (ATU) respectively. In addition, Queen Astrid hospital in Belgium follows the magistral phage approach at the request of the patients. Due to the lack of a definitive organizational procedure, cPT becomes a time-consuming and costly practice that hinders a patients or guardians motivation to seek phage therapy. However, the increasing spread of antimicrobial-resistant pathogens and the lack of new drugs in the antibiotics pipeline have caused a revival of the use of bacteriophages to treat bacterial infections. There are several reports of cPT since the discovery of phages and most of them have been published in the last 10 years (Supplementary Table S1). In these published reports, cPT has been used mainly to treat *S. aureus*, *P. aeruginosa,* and *E. coli*, and to a lesser extent *Enterococcus* spp. induced infections of different body organs. However, there is a need to develop a relatively standardized protocol to drive the phage therapy beyond its compassionate use to a more accessible frontline therapy.

## The immune responses role

**Figure 3:**
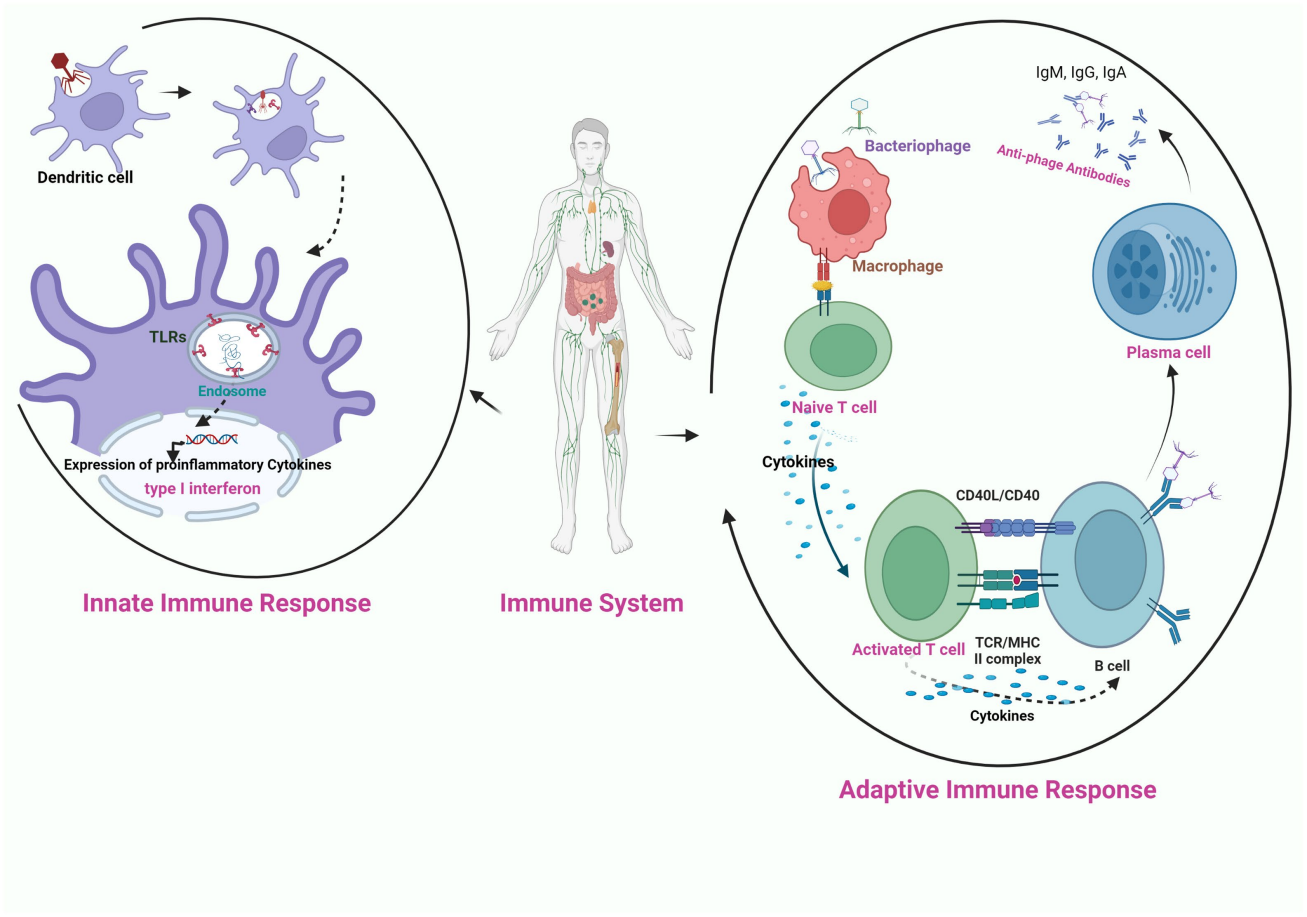
Schematic illustration of the interaction of bacteriophage nucleic acid with the toll like receptors (TLR) present on the endosome of phagocytic cell (innate immune response), activation of pro-inflammatory cytokines and production of anti-phage antibodies through the activation of adaptive immune response on exposure of phage to APC (antigen presenting cell).

The success of phage therapy is influenced by the innate and adaptive immune cells that are provoked by the phages upon their administration in the body. Among the immune interactions, the primary determinant is the recruitment of phagocytes at the infection site through immune recognition *via* pattern recognition receptors (Roach et al., 2017). Differences in immune cell activation are mainly observed based on the type of phage and phage dose. Only a few studies have been conducted on the anti-phage humoral response during phage treatment (Kucharewicz-Krukowska and Slopek, 1987; Bruttin and Brüssow, 2005; Górski et al., 2007; Łusiak-Szelachowska et al., 2014). Biswas et al. (2002) observed that a single intraperitoneal injection of the high dose of lytic phage rescued 100% of the bacteremic mice with no adverse events due to the production of phage-neutralizing antibodies even upon multiple injections of the phage. Secondly, the success of phage therapy depends on the duration for which phages circulate in the body. The rapid eradication of bacteriophages from the body due to the production of the antibodies results in the reduction of their effective dose available for infecting bacteria. In this regard, Merril et al. (1996) reported that mutation in the capsid E protein enabled the lambda phage from escaping the entrapment of the reticuloendothelial system and their long circulation rescued the bacteremic mice. The immune response can be potentially triggered by the externally presented phage proteins (Van Belleghem et al., 2017; Figure 3). Therefore, immunogenicity must be considered while screening phages for therapy and avoiding formation of phage-neutralizing antibodies. Kim et al. (2008) observed that conjugation of the monomethoxypolyethyleneglycol with the phages enhanced the efficacy of the therapy by increasing the survival of infective phages by decreasing their susceptibility to the innate and adaptive immune responses.

Many studies have suggested that there is a correlation between the route of phage administration and anti-phage antibody production. Ochs et al. (1971) studied the immunological response of intravenously injected phage varphiX174 in 26 patients suffering from immunodeficiency syndrome. No antibody response was found in eight patients. However, among the remaining 18 patients, anti-phage IgM antibodies were observed in 10 patients and in all other patients, both IgM and IgG antibodies were detected to clear out the phage. In another study, only 54.4% of patients showed an increase in anti-phage antibody production during phage therapy (Kucharewicz-Krukowska and Slopek, 1987). Khatami et al. (2021) reported that intravenous treatment with phage preparation initially increased the innate immune response in a 7-year-old child with chronic *P. aeruginosa* osteoarticular infection and after only a few days of treatment, there was an increase in the adaptive immune response. However, it was not much significant, as it did not interfere with the therapy. Similarly, no significant up-regulation of immune response was reported *in vivo* (Bruttin and Brüssow, 2005; Górski et al., 2007; Żaczek et al., 2016) or *in vitro* studies (Hodyra-Stefaniak et al., 2015; Majewska et al., 2015).

Łusiak-Szelachowska et al. (2014) have reported very low rate of phage inactivation in oral and oral/local administration route of phage delivery. Similarly, Majewska et al. (2015) observed that T4 phage given orally at high doses to mice showed no change in antibody production in the first 2 weeks, followed by an increase in blood serum IgG within weeks 3–5. Until days 63–79, serum IgA did not increase, but when it reached its maximum, it antagonized the gut transit of active phages, and no phage particles were found in the feces.

Hodyra-Stefaniak et al. (2015) observed the rise of IgG and IgM antibiodies in the sera of a murine systemic inflammatory response syndrome (SIR) model upon subcutaneous administration of *Pseudomonas* phages F8 and T4. Currently, there is no information about the role of phage-specific factors on phage clearance. In addition, a limited number of reports of the use of virulent phages in clinical studies put a gap in our understanding of phage immune interactions.

## Effect of phage therapy on the human gut microbiome

Microbial cells originating from all three domains of life find harbor in the human body; among them, the bacteria and bacteriophages outnumber the eukaryotic human cells, and they are present within the human eye, oral and nasal cavity, gut, and genital tract. Phages infecting the Actinobacteria, Bacteroides, *Lactobacillus* spp.*, Lactococcus* spp., *Listeria* spp.*, Streptococcus* spp.*, Staphylococcus* spp.*, Eubacterium* spp.*, Escherichia* spp., and *Klebsiella* spp. are the most predominant components of the gut virome (Dalmasso et al., 2014). Most bacteriophages in the intestine exist as temperate phages. Furuse et al. (1983) reported 56.6% temperate and 2.5% virulent phages in the fecal sample collected from healthy individuals. Also, while in patients suffering from digestive or respiratory diseases, the frequency of virulent phages increased as compared to healthy individuals, however temperate phages were still predominant in their fecal samples. Moreover, phages that were predominant in the intestine were unique to each individual. Caudovirales present in the healthy individuals gut mucosa showed lower abundance with higher richness and diversity compared to patients suffering from ulcerative colitis (UC) (Zuo et al., 2019). Therefore, a requisite balance between the lytic and lysogenic phages is reported to be essential to maintain human health. An increase in prophage activation resulting in the release of bacterial debris and causing inflammation in patients suffering from inflammatory bowel disease was reported (Manrique et al., 2017). Similarly, Tetz et al. (2017) reported that oral administration of commercial phage cocktails against *Enterobacteriaceae*, *Staphylococcaceae*, *Streptococcaceae*, and *Pseudomonadaceae.*

Caused an increase in gut mucosa permeability in a rat model due to inflammation caused by phage lysate, which targeted the host community and resulted in microbiome composition changes in the intestine. In a report by the Wyss Institute at Harvard, the administration of therapeutic phages leading to modulation of the gut metabolome from the co-colonization of non-susceptible commensal bacteria and transfer of virulent genes between bacteria through horizontal gene transfer was reported (Hibbing et al., 2010).

Similarly, De Sordi et al. (2017) observed the co-evolution of phages in the gut where a single amino acid substitution within the tail fiber protein of P10 phage, resulted in the expansion of the lytic spectrum from *E. coli* LF82 to *E. coli* MG1655 as well. Ott et al. (2017) observed that the transfer of the sterile fecal filtrate containing metabolites, bacterial components, and bacteriophages from healthy individuals to patients suffering from recurrent *Clostridium difficile* infection (CDI) restored healthy gut function and suggested fecal filtrate transfer (FFT) as an alternative and effective approach in comparison to fecal microbiota transplantation (FMT). Similarly, Kao et al. (2019) observed that lyophilized fecal microbiota transplantation (LFMT) in patients suffering from recurrent *C. difficile* infection (RCDI) eliminated the symptoms of infection and restored a healthy gut microbiome.In a recent report by Mu et al. (2021), no change in the gut microflora of a patient during 1 month of phage therapy was observed. However, despite being a predominant component of the gut microbiome, there is still sparse knowledge about the actual effects of phages on gut modulation. There are remarkable gaps in the recognition of phage-host interactions in the gut and how their interactions shape the microbiome communities *in vivo*. So far, most of the phage therapy studies are based on single phage-host pairs or a cocktail of a limited number of phages against the host *in vitro*. Therefore, understanding the phage interactions with their host and immune response *in vivo* lays the first stepping stone for the oral therapeutic application of phages in humans.

## Phage applications in the food industry

Foodborne illness due to bacterial contamination continues to be a significant food safety issue throughout the world. According to Scharff (2012), community health and the annual economic cost of foodborne bacterial infections lead to more than $75 billion loss in United States. The foodborne microbes lead to product loss at the time of manufacturing by recalling of contaminated products back to the food industry. Thus, there is a strong need to develop novel approaches for preventing bacterial pathogens from contaminating a broad range of food products and providing a safe food supply. Food animals are asymptomatic carriers of pathogens. These pathogens can spread from one animal to another, to slaughterhouses and through the food processing facilities to the food items, and finally to the consumer. According to a study published by the World Health Organization in 2010, due to pathogenic bacterial infection, globally around 350 million illnesses and 187,000 deaths occur (Havelaar et al., 2015). Amongst bacterial pathogens *E. coli*, *Campylobacter* spp., non-typhoidal *Salmonella enterica*, *Shigella* spp., *Vibrio cholera,* and *Listeria monocytogenes* were responsible for 96% of the food-borne illnesses. The food industry routinely utilizes several antimicrobial interventions such as chemicals, physical disruption techniques, and irradiation to eradicate the pathogens of the contaminated foods (Maukonen et al., 2003). In the case of fresh fruits, vegetables, and ready-to-eat food products, harsh chemicals, while in liquid and dairy products, heat pasteurization and high-pressure processing (HPP) methods are used to reduce bacterial load (Sohaib et al., 2016). However, these antimicrobial approaches have some significant drawbacks, such as chemicals corroding the food processing equipment and toxicity issues of elements that are not environment or consumer-friendly. On the other hand, heat pasteurization and HPP reduce the nutritional value of some foods.

Therefore, there is a need to identify environment-friendly antimicrobial approaches such as phage biocontrol under this category. While we are talking about phage biocontrol, it means using lytic bacteriophages to eradicate foodborne bacterial pathogens and to make the foods safe for consumption. Due to their specificity to the host, phages solve the problem associated with the traditional decontamination strategy and support the development and commercialization of bacteriophage-based products (Supplementary Table S2) in the food industry (Moye et al., 2020). Regarding regulation of phage-based biocontrol in food-grade, phage products undergo endotoxin testing to get the GRAS (Generally Recognized as Safe) status provided by FDA. Endotoxin levels in final products must be less than 250,000 EU/mL for market regulation; products with higher concentrations will not be granted the GRAS status. According to the GRAS approval, only 10^8^ PFU of phage application is permitted per gram of food, which is just a minute amount of the naturally available phage. The cost of non-phage antimicrobial methods ranges from 10–30 cents per pound, while in the case of phages; it ranges from 1–4 cents per pound of treated food. Hence, phage-based approaches are cheaper than the currently available antimicrobial interventions (Viator et al., 2017). Secondly, most phage-based products (e.g., EcoShield™) do not contain any additives or preservatives. Therefore, phage biocontrol is a consumer-friendly, green and safe approach for the containment of food pathogens.

## Phage-mediated biocontrol of important pathogens of aquaculture

Fisheries and aquaculture sectors are essential elements of the food industry. The largest seafood exporting countries on a global scale are China, Norway, Vietnam, India, and Chile. Global aquaculture production was estimated to be 114.5 million tonnes in 2018, with more expected in the coming year (FAO, 2020). Seafood constitutes the decisive diet in many world areas, as it is affluent in proteins, vitamins minerals, omega-3 fatty acid docosahexaenoic acid (DHA), and eicosapentaenoic acid (which help regulate heart-related problems and Alzheimer’s disease). The threat of microbial contamination continuously challenges the fisheries sector and fish farms and hatcheries are at consistent risk of microbial eruption. *Aeromonas* spp., *Edwardsiella* spp., *Flavobacterium* spp., *Renibacterium* spp., *Streptococcus* spp., *Vibrio* spp., and *Yersinia* spp. (Sudheesh et al., 2012) are the most common fish pathogens that can voluntarily intrude the tissues and skin of aquatic food animals, causing spoilage by forming toxic chemical compounds like trimethylamine, ammonia, H_2_S, and indole (Erkmen and Bozoglu, 2016). It has been reported that in the United States alone, about 84,000 people get a foodborne infection from *Vibrio* spp. While only a few antibiotics have been approved for application, some are used regularly in aquaculture for disease prevention and growth promotion. The overuse of antibiotics has resulted in the emergence of antibiotic-resistant food-borne pathogens (Schar et al., 2021). Along with this, several studies support the presence of antimicrobial residue in aquaculture products. Therefore, several countries have made regulations for the control of antibiotic overuse. However, the stringent antimicrobial regulations influence the import and export of aquaculture products. For example, in response to antimicrobial regulation, Thai shrimp export fell from 24 to 5% in 4 years (Holmström et al., 2003) and the consequences of different antimicrobial regulations in different countries resulted in the ban of seafood products from the importers countries. Therefore, there is a need for biosecurity measures to tackle these problems, and one of the measures includes the incorporation of bacteriophages for biocontrol. For the application of phage therapy, water parameters like temperature, pH, salinity, dissolved organic matter content, and dissolved oxygen are important parameters for consideration. Small tanks are always at greater risk due to the high density of fish. It was observed that phages against *Y. enterocolitica* lysed their host at low temperatures due to the inactivity of phage receptor protein OmpF of *Y. enterocolitica*, at high temperatures (Leon-Velarde et al., 2016). Similarly, the pH changes affected the activity of *F. psychrophilum* phage (Akhwale et al., 2019). Water salinity is also an important factor. Choudhury et al. (2019) observed the activity of *V. harveyi* phage at three different salinities and found that salinity of 25 ppt is more favorable for phage activity. Kalatzis et al. (2016) observed that the phage against *V. alginolyticus* was efficient only at high multiplicity of infection (MOI). Similarly, Kim et al. (2015) used phage PAS-1 at high MOI to obtain a significant effect on rainbow trout. But different results were obtained in the *in vitro* experiment of phages against *A. salmonicida,* which showed significant lysis at low MOI in comparison with high MOI (Chen et al., 2018). Almeida et al. (2019) in their study showed the prophylactic value of phages by observing that an applied dose of phage lysate improved the health conditions of fish by reducing the infections caused by *F. columnare*. In juvenile *Senegalese sole*, the application of phages against *A. salmonicida* only moderately affected the fishs intestinal bacterial community (Silva et al., 2016). Similarly, it was observed that phages in fishes balanced the microbial profile of their gut and modulated their immunity (He and Yang, 2015). With the commercially available phage cocktail (BAFADOR) against *Aeromonas* spp. and *Pseudomonas* spp., it was observed that along with its antibacterial action, the phage cocktail increased the levels of immunoglobulins, protein, and lysozyme and improved the activity of spleen phagocytes as well, thus showing immunomodulatory activity in rainbow trout (Schulz et al., 2019). Similar results were observed on *A. hydrophila* and *P. fluorescens* infected European eels after applying BAFADOR (Schulz et al., 2019). Thus, phages can work as a promising alternative to antibiotics in the fish and seafood industry by imposing their lytic action against infection-causing pathogens simultaneously serving as an immunomodulator. Table 2 represents a summary of the important attempts made for the biocontrol of aquaculture pathogens with the help of phages.

**Table 2:** Summary of phage biocontrol in aquaculture.

Target bacteria	Animal model	Phage	Mode of application	Result	References
*Lactococcus garvieae*	*Seriola quinqueradiata* (Yellow tail)	PlgY-16 (*Siphoviridae*)	Intraperitoneal and oral	The survival rate was much higher in phage treated yellowtail compared to the control group	Nakai et al. (1999)
*P. plecoglossicida*	*Plecoglossus altivelis* (Ayu fish)	PPpW-3 (*Myoviridae*) and PPpW-4 (*Podoviridae*)	Oral	The survival rate increased in phage treated group compared to control group	Park et al. (2000); Nakai and Park (2002), Park and Nakai (2003)
*A. salmonicida*	*Oncorhynchus fontinalis* (Brook trout)	HER110 (*Myoviridae*)	Immersion	Delay in the infection of bacteria and phage treatment reduced bacterial concentration from 10^8^ CFU/mL to 10 CFU/mL in 3 days	Imbeault et al. (2006)
*S. iniae*	*Paralichthys olivaceus* (Japanese flounder)	Phage cocktail	Intraperitoneal	The mortality of phage treated fish reduced significantly compared to the control group.	Matsuoka et al. (2007)
*F. columnare*	*Clarias batrachus*	Nine phage FCP1-FCP9 (*Podoviridae)*	Intramuscular, oral and immersion	Phage treatment increased the survival rate up to 100%	Prasad et al. (2011)
*F. psychorphilum*	*Oncorhynchus mykiss* (Salmo salar)	1H, 6H, 9H,2P, 23 T, FpV9 (*Siphoviridae*) and FpV4, 2A (*Podoviridae*)	Intraperitoneal	A 16–100% reduction in mortality after phage treatment	Castillo et al. (2012)
*V. harveyi*	*Litopenaeus monodon* (Black tiger shrimp)	VHP60 (*Siphoviridae*)	Immersion	Phage treatment improved survival of shrimp post-larvae by 40–60%	Raghu Patil et al. (2014)
*V. parahaemolyticus*	Oyster	pVp-1 (*Siphoviridae*)	Immersion	Phage application on the surface of oysters reduced the bacterial growth to its minimum level	Jun et al. (2014)
*A. salmonicida* subspp.	*Oncorhynchus mykiss* (Rainbow trout)	PAS-1 (*Myoviridae)*	Intramuscular	A total of 26.7% increase in survival rate after phage treatment	Kim et al. (2015)
*A. salmonicida*	Senegalese sole	AS-A (*Myoviridae)*	Immersion	After 6 h of phage treatment, bacterial growth was inhibited in juvenile fish. After 72 h of phage treatment, no morality compared to the control group (mortality 36%) was recorded	Silva et al. (2016)
*V. splendidus*	*Apostichopus japonicas* (Sea cucumber)	PVS-1, and PVS-2 (*Myoviridae)* PVS-3 (*Siphoviridae*)	Diet supplemented with individual phage or cocktail	Survival rate was 82% in phage cocktail treated group compared to single phage and control group	Li et al. (2016)
*V. parahaemolyticus*	*Mytilus edulus*	VP10 phage cocktail	Immersion	1.3 × 10^3^ PFU /mL of VP10 phage cocktail reduced bacteria to undetectable level in mussels	Onarinde and Dixon (2018)
*S. agalactiae*	*Oreochromis miloticus* (Nila tilapia)	HN48	Phage preparation added to the tank	A 60% survival rate compared to control group	Luo et al. (2018)
*V. parahaemolyticus*	*Penaeus vannamei* (Shrimp)	pVp-1 *(Siphoviridae)*	Immersion and fed with phage containing pellet	Mortality was reduced from 100% to 25–50% in phage treated juvenile shrimp	Jun et al. (2018)
*V. anguillarum*	*Gadus morhua* (Atlantic cod)	KVP40 *(Myoviridae)*	Immersion	Phage treatment reduced the mortality rate	Rørbo et al. (2018)
*Vibrio spp.*	*Litopenaeus vannamei* (Shrimp)	Phage cocktail ValLY-3, VspDsh-1, VspSw-1, VpaJT-1, and ValSw4-1 (*Siphoviridae)*	Immersion	Survival rate increased up to 91.1% in phage treated shrimp compared to the control group	Chen et al. (2019)
*A. hydrophila*	*Misgurnus anguillicaudatus*	AKH-2 (*Siphoviridae*)	Immersion	Loach treated with phage showed improvement in survival rate	Akmal et al. (2020)

## Current challenges and future perspectives of phage-based therapy and biocontrol

Despite being the most abundant entities present on earth (10^31^), a very limited number of phages (~15,000) have been characterized completely (Cook et al., 2021), and amongst these, only a few have been utilized so far for therapeutic applications. Improvements in laboratory culturing techniques, use of metagenomics, and *in silico* tools such as PHASTER, VirSorter, MARVEL, and Virnet, etc. for mining the phage sequences in datasets have become essential for identifying novel phage genes and genomes (Rosario and Breitbart, 2011; Khot et al., 2020). Nearly 30% of the identified phages in the databases are of temperate nature, thus restricting their use in therapy due to the problem of horizontal gene transfer (Cook et al., 2021). Also, the lytic range of phages isolated from natural environments rarely covers the large spectrum of clinically associated bacteria. The host specificity in bacteriophages, which on one hand is beneficial in terms of being harmless to normal gut microflora, on the other hand, it poses a hurdle where phages have to be isolated and used against a multitude of rapidly evolving resistant bacterial strains. Thus, isolating a new bacteriophage every time for a new infection will significantly slow down the treatment process. However, these limitations can be overcome by using more than one way. The use of polyvalent phages, which have receptors for binding to more than one kind of bacterial strain or species (Sui et al., 2021), may be employed. Various isolation and enrichment techniques have been devised to yield such broad spectrum phages (Yu et al., 2016). On the other hand, rather than using an individual phage, a cocktail comprising bacteriophages with diverse host ranges can be effective in targeting different bacterial infections at a time. Phage cocktail BFC-1 eradicated musculoskeletal infections caused by *Staphylococcus*, *Pseudomonas*, and *Enterococcus spp.* (Onsea et al., 2019). Another approach is the targeted chemical or genetic modification of the phages to improve their suitability for therapy. Chemical methods of modification of phage structure and function involving the use of chemicals such as silver nanoparticles (AgNPs), luminogens with aggregation-induced emission properties (AIEgens), PheophorbideA (PPA) and Indium Tin Oxide (ITO), etc., can also be used. Genetic modification of phages involves genetic mutation, gene replacement, and integration of foreign genes using molecular techniques such as traditional homologous recombination-based techniques, bacteriophage recombineering of electroporated DNA (BRED), and CRISPR-Cas based phage engineering (Wetzel et al., 2021). Gene mutation and gene replacement have been mainly carried out in the receptor-binding protein-encoding genes (Yosef et al., 2017) or those regulating replication mechanisms. While, in the case of gene integration, a foreign gene was integrated into the non-functional region of the phage genome to improve the phage activity in biofilms and modification of the temperate phage to become lytic (Dedrick et al., 2019). Use of bacteriophage encoded lysins, which are the proteins responsible for peptidoglycan hydrolysis and subsequent host lysis, has been used to target even multiple genera of bacteria. Chimeric lysins or chimeolysins, created by domain shuffling from natural lysins and artilysins are created by fusion of endolysin with other components from a peptide or a protein (Yang et al., 2015; Defraine et al., 2016). Yang et al. (2015) have reported a chimeolysin ClyR with lytic activity on three genera, including *Streptococcus*, *Staphylococcus* (including MRA and VRSA), and *Enterococcus*. Bacteriophage or lysins induced cell lysis in the case of gram-negative bacteria may lead to the release of endotoxins which are responsible for inflammatory response and, in severe cases, may cause septic shock. While the use of another phage-derived enzyme, polysaccharide depolymerase helps to overcome this problem because, as such, they do not lyse the bacterial cell but only remove the surrounding polysaccharide layer, thus exposing the bacteria to immune cells (Azeredo and Sutherland, 2008).

The efficacy of phage therapy is also limited by the selection of phage-resistant bacterial strain due to prolonged treatment with bacteriophages. The use of bacteriophage cocktails and their combined application with other antimicrobial agents like antibiotics or nanoparticles has been shown to suppress the evolution of resistance. Applying different selective pressures have been suggested to be more effective than individual ones in reducing bacterial growth as well as in controlling the evolution of resistance (Gelman et al., 2018). The use of phage lysins as antibacterials is associated with quick lysis action and minimal risk of resistance development as they target peptidoglycan components, which are essential for the survival of bacteria.

Moreover, the lack of interest of the pharmaceutical sector towards phage-based products due to associated patentability issues has also impeded phage research globally. The use of phages as counter-medicine against antibiotics could be seen as economically undesirable for the established drug manufacturing industries. The existing regulatory guidelines for the approval of antibacterial agents are designed in accordance with the development of chemical-based antibiotics, which are, as such, unsuitable for phage therapy or its approval in medicine. But, as biological therapeutic proteins, phage-derived products such as endolysins, polysaccharide depolymerase or genetically modified phages are appropriate for approval under existing procedures. Moving further in terms of practical involvement of phages as therapeutic agents in clinics or as biocontrol in industry, the gaps in knowledge about phage biology, specificity, pharmacodynamics, pharmacokinetics, and immunomodulatory effects, need to be addressed by intense investigative studies. As the phages are pervasive, the human immune system does not stay naïve to them. Therefore, to avoid any treatment failure, synergy assessment (in the case of phage cocktails/phage-lysin combinations/phage-antibiotic combinations) and immune reaction studies should be carried out extensively. The regulations involved in phage preparation and the legal framework should be set up decisively. Also, there is a need to formulate universal and favorable regulations to promote phage or phage-based products for human benefits. In addition, popularizing phage therapy and its benefits over other therapeutic agents will help in the acceptance of phage therapy by medical practitioners. Awareness should be generated among clinicians to offer phage therapy as a treatment option for patients where antibiotics have failed altogether. The areas that need to be strengthened, of course, include expanding phage biobanks/repositories for the timely offering of specific and usable phages, improvements in phage production protocols to provide stable and safe phage preparations, and to relax the regulatory protocols for phage therapy. Looking at the extensive damage to the environment, animal, and human health that antibiotics have posed since their discovery, and simultaneously the benefits that the naturally occurring phages (the living drugs) have offered, it does not seem relevant to completely reject phages.

## Conclusion

The inclusion of bacteriophages in the treatment of human diseases and food biocontrol has witnessed a significant surge in the last few decades. A large number of studies employing single phages, cocktails, phages in combination with antibiotics, and simultaneous improvements in phage production protocols and the ease of genetic manipulation technologies, have broadened the versatility of phage application. However, there is a requirement for the adoption of favorable regulations to promote phage or phage-based products for human/livestock benefits. The phage therapy provides hope against ever growing menace of antimicrobial resistance however, a major boost is required for widening its application through the involvement of researchers, clinicians, industry, and policy makers. Use of phage therapy also aligns with the goal of one health approach to sustainably benefit the environment with simultaneous improvement in the treatment strategies. Incorporation of phage therapy in medical education and willingness among physicians to consider and apply phages will help accelerate its acceptance. Further, making phage therapy cost-effective by supporting medical tourism and relaxing the stringent regulatory guidelines associated with its compassionate use will help in improving the accessibility of phage therapy as a frontline medical intervention to treat resistant bacterial infections.

## Author contributions

AJ and TA: conceptualization. AJ: methodology and software. AJ, MV, TA, and RV: validation. NV, BB, RV, and BT: formal analysis. TA: resources and funding acquisition. AJ and MV: data curation. AJ and RV: writing - original draft. MV, TA, NV, and BB: writing - review and editing. AJ, MV, and RV: visualization. TA and BT: supervision. TA and RV: project administration. All authors have agreed to the published version of the manuscript.

## Funding

This work was supported by the National Agricultural Science Fund, Indian Council of Agricultural Research, New Delhi, India and Council of Scientific and Industrial Research, New Delhi, India.

## Conflict of interest statement

The authors declare that the research was conducted in the absence of any commercial or financial relationships that could be construed as a potential conflict of interest.
