# Supplementary material for: Tracking the phage trends: A comprehensive review of applications in therapy and food production
Source: Front Microbiol. 2022 Nov 24;13:993990. doi: 10.3389/fmicb.2022.993990 (PMC9730251; doi:10.3389/fmicb.2022.993990)
Supplement: Supplementary file 1 [file Table_1.docx]

Table 2: Summary of the Compassionate use of phage therapy since 2011

| **Infection** | **Pathogens** | **Phage** | **N** | **Mode of application** | **Results** | **Phage source/Company/Institution** | **References** |  |
| --- | --- | --- | --- | --- | --- | --- | --- | --- |
| Urinary tract infection | *P. aeruginosa* | Pyophage | 1 | Local | Phage and antibiotics synergistically eradicate the pathogenic bacteria | Georgia Eliava Institute of Bacteriophages in Tbilisi, Georgia | Khawaldeh et al., 2011 |  |
| Burn wound | *P. aeruginosa, S. aureus* | BFC-1 (Cocktail of  *Siphoviridae&*  *Myoviridae)* | 9 | Topical | No adverse effect, the bacterial infection was not completely eradicated | Military | Rose et al., 2014 |  |
| Corneal infection | *S. aureus* | SATA-8505 (Not described) | 1 | Topical, Intravenous, Nasal spray | Improvement of the ocular signs and eradication of bacterial infection both from the eye and nasal route | Phage Therapy Center, Tbilisi, Georgia | Fadlallah et al., 2015 |  |
| Diabetic toe ulcer | *S. aureus* | Sb-1 (Not described) | 6 | Topical | Ulcers healed on average seven weeks and amputation was avoided | Georgi Eliava Institute of Bacteriophages in Tbilisi, Georgia, | Fish et al., 2016 |  |
| DiGeorge Syndrome, Bacteremia | *P. aeruginosa* | Phage cocktail  (Not described) | 1 | Intravenous | Eradication of bacteremia with the first six doses of phage cocktail, but subsequent regrowth occurred and the patient’s condition worsened due to cardiac failure | Military | Duplessis et al., 2017 |  |
| Acute kidney injury | *P. aeruginosa* | BFC-1 (Cocktail of *Siphoviridae &*  *Myoviridae)* | 1 | Intravenous, local | Blood culture turned negative, CRP level dropped, fever disappeared, No adverse effect | Military | Jennes et al., 2017 |  |
| Necrotizing pancreatitis in Diabetic patient | *A. baumannii* | Phage cocktail AB-Navy1, AB-Navy4, AB-Navy71, & AB-Navy97 (*Myoviridae*) AbTP3Φ1 (*Podoviridae*), C1P12, C2P21, C2P24 & AC4 (*Myoviridae*) | 1 | Intravenous, intracavitary | Improvement in health and complete eradication of bacteria, No adverse effect | Environmental sample and sewage water | Schooley et al., 2017 |  |
| Netherton syndrome | *S. aureus* | Sb-1 phage,  Pyophage, Fersis bacteriophage | 1 | Topical, Oral | Hyperaemic area became more minor, mobility improved and area of normal skin began to appear | Eliava Biopreparations Ltd. Gorgia | Zhavania et al., 2017 |  |
| Prosthetic (Aortic valve graft) | *P. aeruginosa* | OMKO1 (Myoviridae) | 1 | Intravenous, direct by fistula | Complete eradication of bacteria from the site of infection | Academic | Chan et al., 2018 |  |
| Craniectomy site | MDR *A. baumannii* | Phage cocktail (Not described) | 1 | Intravenous | No sign of infection, blood culture turned negative | Military | LaVergne et al., 2018 |  |
| Bone and joints | *P. aeruginosa* | 1450 & 1777 (*Myoviridae*) 1792 &1797 (*Podoviridae*) | 1 | Local | Phage and antibiotics synergistically eradicated the bacteria | Biotech | Ferry et al., 2018 |  |
| Prosthetic  Joints | *S. aureus* | Bacteriophage cocktail (1493,1957 and 1815)  (Family not described) | 3 | Local | Phage action on biofilm increased the sensitivity of bacteria to antibiotics | Biotech | Ferry et al., 2018 |  |
| Cystic fibrosis | *Achromobacter xylosoxidans* | Phage cocktail contain two bacteriophage belong to the *Siphoviridae* family | 1 | Inhalation | Improvement of lung function and decrease in the dependency on antibiotics | Environment | Hoyle et al., 2018 |  |
| Urinary tract infection | *E. coli, Proteus* spp*., S. aureus, P. aeruginosa, Streptococcus* spp. | Pyo, Intesti, Ses and Enko Phage | 9 | Local | Bacterial load decreased to a modest level in 67% of the cases | Eliava Bio Preparations Ltd., Tbilisi, Georgia | Ujmajuridze et al., 2018 |  |
| Bone, Gastrointestinal tract, ENT, Urogenital | *E. coli, Proteus* spp.*, S. aureus, P. aeruginosa,* | Phage preparation (Not described) | 15 | Local, oral, intrarectal | Eradication of the pathogenic bacteria | Microgen, (Russia) and the Eliava Institute Georgia | Patey et al., 2019 |  |
| Left tibial infection | XDR *A. baumannii*  *K. pneumoniae* | φAbKT21phi3 *(Autographivir*idae), φKpKT21phi1 (*Drexleviridae*) | 1 | Intravenous | Blood culture turned negative, wound healing and no pain. | Military | Nir-Paz et al., 2019 |  |
| Ventilator associated pneumonia and empyema | *P. aeruginosa* | AB-PA01 (Cocktail of phage belonging to *Myoviridae* & *Podoviridae*) | 1 | Intravenous, nebulizer | Phage and antibiotics synergistically eradicated the infection | AmpliPhi Biosciences | Maddock et al., 2019 |  |
| Left ventricular assist device | *S. aureus* | AB-SA01 (Not described) | 1 | Intravenous | Combined treatment of phage and antibiotics resulted in negative sternal wound and intra-operative samples | AmpliPhi Biosciences | Aslam et al., 2019 |  |
| Urinary tract infection | *K.*  *pneumoniae* | Phage preparation (Not described) | 1 | Oral and bladder istillation | Combined treatment of phage and antibiotic prevented the recurrence of infection | Georgi Eliava Institute of Bacteriophages in Tbilisi, Georgia | Kuipers et al., 2019 |  |
| Disseminated Infection | *M. abscessus* | Muddy, ZoeJ, and BPs (*Siphoviridae*) | 1 | Topical | Reduced bacterial load | SEA-PHAGES | Dedrick et al., 2019 |  |
| Prosthetic joint of knee & chronic osteomyelitis of femur | *P. aeruginosa* | Phage preparation(Not described) | 1 | Local, intravenous | Clearance of MDR *P. aeruginosa* from the body | Georgia Eliava Institute of Bacteriophages in Tbilisi, Georgia, | Tkhilaishvili et al., 2019 |  |
| Musculo-skeletal | *Staphylococcus* spp*., P. aeruginosa, Enterococcus faecalis* | BFC-1 ( Cocktail of *Siphoviridae &*  *Myoviridae)* | 4 | Local | Out of 4 patients, 3 patients became infection-free | Queen Astrid Military Hospital (QAMH) Brussels | Onsea et al., 2019 |  |
| Chronic Rhinosinusitis | *S. aureus* | AB-SA01 (Not described) | 9 | Intranasal | Out of 9 patients, complete eradication of bacteria occurred in 2 patients while in the remaining 7 patients bacterial load was reduced | Ampliphi  Bioscience | Ooi et al., 2019 |  |
| Gastrointestinal, Urinary tract | *K. pneumoniae* | Phage preparation (Not described) | 1 | Oral & intra-rectal | Blood, urine, and stool sample turned negative | Georgi Eliava Institute of Bacteriophages in Tbilisi, Georgia, | Corbellino et al., 2020 |  |
| Prosthetic joint infection | *S. aureus* | SaGR51φ1 (Family not described) | 1 | Intravenous | Eradication of bacteria with no other adverse effect | Military | Doub et al., 2020 |  |
| Urinary tract infection | *K. pneumonia* | Phage cocktail I (SZ-1, SZ-2, SZ-3, SZ-6, and SZ-8), Phage cocktail II (Kp165, Kp166, Kp167, Kp158, and Kp169), Phage cocktail III (Kp152, Kp154, Kp155, Kp164, Kp6377, and HD001) (Family not described) | 1 | Bladder instillation | Combined treatment of phage and antibiotics reduced the infection while their individual treatment does not show efficacy | Shanghai Institute of Phage | Bao et al., 2020 |  |
| Lung | *A. baumannii* | ɸAb124 (*Podoviridae*), ɸAb121 (*Myoviridae*) | 4 | Nebulizer | Phage treatment eradicated the pathogen and improved the patient’s conditions | Shanghai Institute of Phage | Wu et al., 2021 |  |
| Prosthetic infection, Lung infection, Chronic vascular Graft infection, Repetitive treprostinil Pump infection, Sternal wound abscesses | *S. aureus, Enterococcus faecium*  *P. aeruginosa, K. pneumoniae, E. coli* | CH1,Sa30, KpV15, ECD7, V18, PA5 & PA10 ( *Myoviridae*) SCH1SCH111 & KpV811 (*Podoviridae*) , EnF1 (*Siphoviridae*) | 8 | Local, intraoperative and intranasal | In five patients, infection-causing bacteria not detected further | Wastewater, sewage and clinical | Rubalskii et al., 2020 |  |
| Urinary tract infection | *K. pneumoniae* | Phage preparation (Not described) | 1 | Intra-rectal | Combined treatment of phage and antibiotics eradicated the pathogens | Hirszfeld Institute of Immunology and Experimental Therapy, Poland | Rostkowska et al .2021 |  |
| Prosthetic knee | *K. pneumoniae* | KpJH46φZ (*Podoviridae*) | 1 | Intravenous | Local symptoms and signs of infection were resolved | Environment | Cano et al., 2021 |  |
| Chronic Bacterial Prostatitis | *Staphylococcus* spp.  *E. faecalis*, *Streptococcus* spp. | Pyo, Intesti, and Staphylococcal phage | 1 | Oral, urethral instillations | Eradication of the pathogenic bacteria and prostate size became normal | Georgi Eliava Institute of Bacteriophages in Tbilisi, Georgia, | Johri et al., 2021 |  |
| Prosthetic knee | *S. aureus* | AB-SA01, SaGR51ø1 (Family not described) | 1 | Intra-articular | Blood culture turned negative with no other side effect | Ampliphi Biosciences | Ramirez-Sanchez et al., 2021 |  |
| N stands for Number of patients  UTI –Urinary tract infection | | | | | | | | |

References

Response to Q17- References for Supplementary material

1. Aslam, S. *et al.* (2019) ‘Early clinical experience of bacteriophage therapy in 3 lung transplant recipients’, *American journal of transplantation: official journal of the American Society of Transplantation and the American Society of Transplant Surgeons*, 19(9), pp. 2631–2639.
2. Bao, J. *et al.* (2020) ‘Non-active antibiotic and bacteriophage synergism to successfully treat recurrent urinary tract infection caused by extensively drug-resistant Klebsiella pneumoniae’, *Emerging microbes & infections*, 9(1), pp. 771–774.
3. Chan, B.K. *et al.* (2018) ‘Phage treatment of an aortic graft infected with Pseudomonas aeruginosa’, *Evolution, medicine, and public health*, 2018(1), pp. 60–66.
4. Corbellino, M. *et al.* (2020) ‘Eradication of a Multidrug-Resistant, Carbapenemase-Producing Klebsiella pneumoniae Isolate Following Oral and Intra-rectal Therapy With a Custom Made, Lytic Bacteriophage Preparation’, *Clinical infectious diseases: an official publication of the Infectious Diseases Society of America*, 70(9), pp. 1998–2001.
5. Duplessis, C. *et al.* (2018) ‘Refractory Pseudomonas Bacteremia in a 2-Year-Old Sterilized by Bacteriophage Therapy’, *Journal of the Pediatric Infectious Diseases Society*, 7(3), pp. 253–256. doi:10.1093/jpids/pix056.
6. Fadlallah, A., Chelala, E. and Legeais, J.-M. (2015) ‘Corneal Infection Therapy with Topical Bacteriophage Administration’, *The open ophthalmology journal*, 9, pp. 167–168.
7. Ferry, T. *et al.* (2018) ‘Innovations for the treatment of a complex bone and joint infection due to XDR Pseudomonas aeruginosa including local application of a selected cocktail of bacteriophages’, *The Journal of antimicrobial chemotherapy*, 73(10), pp. 2901–2903.
8. Fish, R. *et al.* (2016) ‘Bacteriophage treatment of intransigent diabetic toe ulcers: a case series’, *Journal of Wound Care*, 25, pp. S27–S33. doi:10.12968/jowc.2016.25.sup7.s27.
9. Hoyle, N. *et al*. (2018) ‘Phage therapy against *Achromobacter xylosoxidans* lung infection in a patient with cystic fibrosis: a case report’, *Research in microbiology*, 169(9), pp. 540-542. <https://doi.org/10.1016/j.resmic.2018.05.001>.
10. Jennes, S. *et al.* (2017) ‘Use of bacteriophages in the treatment of colistin-only-sensitive Pseudomonas aeruginosa septicaemia in a patient with acute kidney injury—a case report’, *Critical Care,* 29(1), pp. 1-3. doi:10.1186/s13054-017-1709-y.
11. Khawaldeh, A. *et al.* (2011) ‘Bacteriophage therapy for refractory Pseudomonas aeruginosa urinary tract infection’, *Journal of medical microbiology*, 60(Pt 11), pp. 1697–1700.
12. Kuipers, S. *et al.* (2019) ‘A Dutch Case Report of Successful Treatment of Chronic Relapsing Urinary Tract Infection with Bacteriophages in a Renal Transplant Patient’, *Antimicrobial Agents and Chemotherapy* *64*(1), pp.e01281-19. doi:10.1128/aac.01281-19.
13. LaVergne, S. *et al.* (2018) ‘Phage Therapy for a Multidrug-Resistant Acinetobacter baumannii Craniectomy Site Infection’, *Open forum infectious diseases*, 5(4), p. ofy064.
14. Maddocks, S. *et al.* (2019) ‘Bacteriophage Therapy of Ventilator-associated Pneumonia and Empyema Caused by Pseudomonas aeruginosa’, *American journal of respiratory and critical care medicine*, 200(9), pp. 1179–1181.
15. Nir-Paz, R. *et al.* (2019) ‘Successful Treatment of Antibiotic-resistant, Poly-microbial Bone Infection With Bacteriophages and Antibiotics Combination’, *Clinical infectious diseases: an official publication of the Infectious Diseases Society of America*, 69(11), pp. 2015–2018.
16. Patey, O. *et al.* (2018) ‘Clinical Indications and Compassionate Use of Phage Therapy: Personal Experience and Literature Review with a Focus on Osteoarticular Infections’, *Viruses*, 11(1), p. 18. doi:10.3390/v11010018.
17. Ramirez-Sanchez, C. *et al.* (2021) ‘Successful Treatment of Staphylococcus aureus Prosthetic Joint Infection with Bacteriophage Therapy’, *Viruses*, 13(6), p. 1182. doi:10.3390/v13061182.
18. Rostkowska, O.M. *et al.* (2021) ‘Treatment of recurrent urinary tract infections in a 60‐year‐old kidney transplant recipient. The use of phage therapy’, *Transplant Infectious Disease*. 23(1), p.e13391. doi:10.1111/tid.13391.
19. Rubalskii, E. *et al.* (2020) ‘Bacteriophage Therapy for Critical Infections Related to Cardiothoracic Surgery’, *Antibiotics (Basel, Switzerland)*, 9(5), p. 232. doi:10.3390/antibiotics9050232.
20. Schooley, R.T. *et al.* (2017) ‘Development and Use of Personalized Bacteriophage-Based Therapeutic Cocktails To Treat a Patient with a Disseminated Resistant Acinetobacter baumannii Infection’, *Antimicrobial Agents and Chemotherapy*, *61*(10), e00954-17. doi:10.1128/aac.00954-17.
21. Tkhilaishvili, T. *et al.* (2019) ‘Bacteriophages as Adjuvant to Antibiotics for the Treatment of Periprosthetic Joint Infection Caused by Multidrug-Resistant Pseudomonas aeruginosa’, *Antimicrobial agents and chemotherapy*, 64(1), pp.e00924-19. doi:10.1128/AAC.00924-19.
22. Ujmajuridze, A. *et al.* (2018) ‘Adapted Bacteriophages for Treating Urinary Tract Infections’, *Frontiers in microbiology*, 9, p. 1832.
23. Zhvania, P. *et al.* (2017) ‘Phage Therapy in a 16-Year-Old Boy with Netherton Syndrome’, *Frontiers in Medicine*, 4, p. 94. doi:10.3389/fmed.2017.00094.
