# Supplementary material for: Tracking the phage trends: A comprehensive review of applications in therapy and food production
Source: Front Microbiol. 2022 Nov 24;13:993990. doi: 10.3389/fmicb.2022.993990 (PMC9730251; doi:10.3389/fmicb.2022.993990)
Supplement: Supplementary file 2 [file Table_2.docx]

Table 3: A summary of direct phage application onto a variety of foods (from the year 2010 to the present) The studies from the year 2001-2009 have been reviewed elsewhere (Hagens and Loessner 2010).

| **Reference** | **Food items** | **Targeted pathogen** | **Phages/product** | **Results** |
| --- | --- | --- | --- | --- |
| Soni and Nannapaneni, 2010 | Raw salmon fillets | *L. monocytogenes* | PhageGuard Listex™ | Reduction of 1.8-3.5 logs bacterial counts after the application of phage at ∼10^8^ PFU/g concentration on the surface of contaminated raw salmon fillets stored at 4/ 22°C. |
| Soni et al., 2010 | Raw catfish fillets | *L. monocytogenes* | PhageGuard Listex™ | Decreased microbial count by 1.4-2.0 logs at 4°C, 1.7-2.1 logs bacterial count at 10°C, and 1.6-2.3 logs bacterial count at 22°C. |
| Ye et al., 2010 | Sprouting mung beans & alfalfa seeds | *Salmonella* spp. | F01, P01, P102, P700, P800, and FL 41  (Caudovirales) | Reduction of the bacteria count by ∼3-6 logs on sprouting mung beans |
| Bigot et al., 2011 | Chicken | *L. monocytogenes* | FWLLm1  *(Myoviridae*) | Decreased *Listeria* counts 1-2 logs on the surface of contaminated food stored at 4°C or 30°C. But, subsequently, bacteria regrew at 30°C. |
| Guenther and Loessner, 2011 | soft cheese | *L. monocytogenes* | A511  *(Myoviridae)* | Reduction of *Listeria* count by 2 logs and the natural microbial community of cheese was not affected. |
| Viazis et al., 2011 | Leafy green vegetables | *E. coli O157:H7* | BEC8  *(Myoviridae)* | Bacterial count decreased by ~2-4 logs after the treatment with phage cocktail at 4, 8, 23, & 37°C, and the addition of essential oil enhanced this effect. |
| Bandara et al., 2012 | Fermented soya bean paste | *B. cereus* | BCP1-1 & BCP8-2 *(Myoviridae)* | Decreased bacterial count, the presence of divalent cations enhanced the phage adsorption resulting in eradication of the bacterial load. |
| Bueno et al., 2012 | Cheese | *S. aureus* | vB_SauS-phi-IPLA35 and vB_SauS-phi-SauS-IPLA88  (*Siphoviridae)* | In the cheese prepared from phage-treated milk, counts of *S. aureus* were decreased significantly as compared to the cheese prepared from untreated milk with no effect on the microbiota of milk. |
| Carter et al., 2012 | Beef and lettuce | *E. coli* O157:H7 | EcoShield™ | Reduction of the bacterial load by ≥ 94% in beef and ∼87% in lettuce. |
| Guenther et al., 2012 | Hot dogs, sliced turkey breast, chocolate milk, egg yolk, and mixed seafood | *Salmonella* spp. | FO1-E2  *(Myoviridae)* | Reduced *Salmonella* count to an undetectable level in various foods within 24-48 hours at 15°C and regrowth occurred after a few days, while in chocolate milk and mixed seafood, the treatment with phage at 8°C reduced bacterial counts below detection level within 24 hours with no regrowth. In egg yolk and assorted seafood initial reduction in *Salmonella* count was observed after phage addition but after a few days regrowth matched to that of control. |
| Soni et al., 2012 | Qeso fresco cheese | *L. monocytogenes* | PhageGuard Listex™ | Reduction in *Listeria* count by ∼3 logs on the surface of experimentally contaminated queso fresco cheese, but regrowth of *Listeria* was observed. |
| Boyacioglu et al., 2013 | Leafy green spinach and lettuce | *E. coli O157:H7* | EcoShield™ | Counts of *E.coli* were decreased by > 2 logs after phage application at both 4 and 10°C. |
| Chibeu et al., 2013 | Roasted beef and turkey | *L. monocytogenes* | PhageGuard Listex™ | *Listeria* counts on contaminated ready-to-eat roasted beef and turkey reduced more effectively with phage treatment at 4°C or 10°C, compared to chemical antimicrobial and subsequent regrowth was observed at both temperatures. Combined treatments with phage and chemical antimicrobials showed similar results. |
| Endersen et al., 2013 | Milk | *Mycobacterium smegmatis* | Six phages  (*Siphoviridae*) | Bacterial counts were reduced by 9 logs in experimentally contaminated milk after 96 hours at 37°C. |
| Ferguson et al., 2013 | Lettuce | *E. coli O157:H7* | EcoShield™ | Application of phage cocktail on the surface of lettuce by spraying led to a sizeable initial reduction (~0.8-1.3 logs) in *E. coli* O157:H7 counts. While their application by dipping did not reduce bacterial count significantly. |
| Hudson et al., 2013 | Beef | *E. coli* | FAHEc1  *(Myoviridae)* | *E. coli* counts were reduced by 2-4 logs at various temperatures on the raw and cooked beef surface after phage application in a concentration-dependent manner but regrowth was observed at a higher temperature. |
| Hungaro et al., 2013 | Chicken skin | *Salmonella* spp. | Five phage cocktail  (*Podoviridae*) | Reduced *Salmonella* count by ~1 log on chicken skin. The results were comparable with 200ppm dichloroisocyanurate, 10 ppm peroxyacetic acid and 2 % lactic acid. |
| Kang et al., 2013 | Chicken skin | *S.* Enteritidis*, S.* Typhimurium | wksl3  (*Siphoviridae)* | Phage treatment reduced *Salmonella* spp. counts by ∼3 logs on chicken skin at 8°C. To test for safety phage cocktails (SS3e, vB_SenS-Ent1, SE2, SETP3, and wksl3) were also administered to mice orally and mice displayed no adverse effects. |
| McLean et al., 2013 | Milk | *E. coli* | EC6 (*Siphoviridae*), EC9 *(Myoviridae)*, EC11 (*Podoviridae*) | At 5-9°C and 25°C, phage treatment eradicated *E. coli* from raw and ultrahigh temperature processed (UHT) milk but regrowth was observed in raw milk. |
| Magnone et al., 2013 | Broccoli, cantaloupe, and strawberries | *E. coli, Salmonella* spp.*, Shigella* | EcoShield™, SalmoFresh™, ShigActive™ | Reduction of the pathogenic bacteria more effectively than chlorine, while their combined treatment showed better results. |
| Spricigo et al., 2013 | Fresh eggs  Lettuce | *Salmonella* spp | UAB_Phi 20, & UAB_Phi78 (*Pdoviridae*), UAB_Phi87 *(Myoviridae)* | Reduction in *Salmonella* counts by ∼1 log on fresh eggs after 60 mins, 2-4 logs on lettuce after 60 mins, 1-2 logs on chicken breasts at 4°C and 2-4 logs on pig skin at 33°C after 6 hours. |
| Zhang et al., 2013 | Spiced chicken | *Shigella* spp | SD-11, SF-A2, SS-92 *(Myoviridae)* | Reduction of *Shigella* spp. count by ∼1-4 logs at 4°C. |
| Galarce et al., 2014 | Salmon fillets | *S. Enteritidis* | Five phages cocktails | Phage cocktail treatment in raw salmon fillets reduced the bacterial count by 2.8- 3.2 logs and in smoked salmon fillets 1.2-1.9 logs after 10 days of storage at 4°C and 18°C, respectively. |
| Oliveira et al., 2014 | Melon, pear and apple slices | *L. monocytogenes* | PhageGuard Listex™ | Phage treatment reduced the *Listeria* count on experimentally contaminated melon and pear slices at 10°C in a dose-dependent manner, but *Listeria* levels in apple slices remained unaffected. |
| Silva et al., 2014 | Soft cheese | *L. monocytogenes* | P100 | Phage treatment initially reduced the *Listeria* count, but after storage for 7 days at 10°C on experimentally contaminated soft cheese, regrowth was observed. |
| Zinno et al., 2014 | Chicken samples, apple juice, liquid egg, energy drink, and skimmed milk | *Salmonella* spp. | P22 (Caudovirales) | *Salmonella* count decreased by 0.5-2 logs, ~3 logs, ~3 logs, ~2 logs, and below the detection limit on the experimentally contaminated chicken samples, apple juice, liquid egg, energy drink, and skimmed milk, respectively, after the treatment with phage and storage at 4°C. |
| Perera et al., 2015 | Cheese, lettuce, smoked salmon, frozen entrèes and apple slice | *L. monocytogenes* | ListShield™ | Reduction in *Listeria* count by 1.1 log, 0.7 logs, 1.0 log, and 2.2 logs on the surface of experimentally contaminated lettuce, cheese, smoked salmon, and frozen entrèes, respectively. Application of phage alone or in combination with an antioxidant/anti-browning solution reduced bacterial count by 1.1 logs on apple slice after 24 hours at 4 °C. |
| Sukumaran et al., 2015 | Chicken breast fillets | *Salmonella* spp. | SalmoFresh™ | Combined treatment with chlorine or PAA and phage to experimentally contaminated chicken breast reduced *Salmonella* count more effectively than alone. |
| Hong et al., 2016 | Ground pork and eggs | *Salmonella* spp. | SJ2 (*Siphoviridae*) | *Salmonella* counts reduced significantly in experimentally contaminated ground pork and eggs after phage treatment. More phage-resistant *Salmonella* colonies were recovered from the egg. |
| Soffer et al., 2016 | Raw pet foods | *Salmonella* spp. | SalmoLyse® | *Salmonella* counts decreased by 60 to 92%. |
| Sukumaran et al., 2016 | Chicken breast fillets | *Salmonella* spp. | SalmoFresh™ | After phage treatment by dipping or surface application to the chicken breast fillet and storage at 4°C significant reductions in *Salmonella*, counts were observed by up to 0.9 logs; storing the meat in modified atmospheric packaging after phage application decreased bacterial count up to 1.2 logs. |
| Figueiredo and Almeida, 2017 | Sliced pork ham | *L. monocytogenes* | PhageGuard Listex™ | Reduction in *Listeria* count to an undetectable level after 72 hours. |
| Grant et al., 2017 | Boneless chicken thighs and legs | *Salmonella* spp. | PhageGuard S™ | Treatment with phage solution prepared with tap water to the experimentally contaminated boneless chicken thighs and legs resulted in a more significant reduction of *Salmonella* count compared to that prepared using filtered water. |
| Soffer et al., 2017 | Lettuce, melon, smoked salmon, corned beef, & pre-cooked chicken | *Shigella* spp. | ShigaShield™ | In various ready-to-eat (RTE) foods, a 90% reduction in *Shigella* counts was observed after phage addition. |
| Hagens et al., 2018 | Skinless and skin-on poultry products | *Salmonella* spp. | PhageGuard S™ | *Salmonella* counts were reduced by more than 1 log on various poultry products. |
| Yeh et al., 2018 | Ground beef | *Salmonella* spp. | PhageGuard S™ | *Salmonella* count reduced by ~1 log on experimentally contaminated ground beef trim after treatment with phage or irradiation individually. However, combined therapy with phage and irradiation reduced bacterial count by ~2 logs. |
| Bai et al., 2019 | Lettuce and cucumber | *Salmonella* spp. | BSPM4 (*Siphoviridae)*, BSP101 *(Myoviridae)*, BSP22A (*Siphoviridae*) | Reduction of *Salmonella* count by 4.7-5.8 logs. |
| Islam et al., 2019 | Chicken breast and milk | *Salmonella* spp. | LPSTLL *(Siphoviridae)*, LPST94 (*Ackermannviridae*), LPST153 (*Podoviridae*) | Reduction of *Salmonella* count below the detection limit on various foods while phage cocktail reduced bacterial count by 5.23 logs on microtiter plates and steel chips, showing their effectivity against *Salmonella* formed biofilms over food processing surfaces. |
| Zhang et al., 2019 | Lettuce and sprouts | *Salmonella* spp. | SalmoFresh™ | *Salmonella* count was reduced by 2-3 logs with phage treatment and by 2.7-3.8 logs using combined treatment with chlorine and phage. |
| Shebs-Maurine et al., 2020 | Beef | *E. coli O157:H7* | MS (Caudovirales) | A 0.7 to 1.3 log reduction of *E. coli* was observed on ground beef. |
| Vikram et al., 2020 | Beef chuck roast, ground beef, chicken breast, cooked chicken, salmon, cheese, cantaloupe, and romaine lettuce | *E. coli O157:H7* | EcoShield PX™ | Bacterial counts decreased by 97% on various food products after the treatment with phage cocktail at two different concentrations (5 × 106, or 1 × 107 PFU/g). |

References

Response to Q17- References for Supplementary material

1. Bai, J., Jeon, B. and Ryu, S. (2019) ‘Effective inhibition of Salmonella Typhimurium in fresh produce by a phage cocktail targeting multiple host receptors’, *Food microbiology*, 77, pp. 52–60.
2. Bandara, N. *et al.* (2012) ‘Bacteriophages BCP1-1 and BCP8-2 require divalent cations for efficient control of Bacillus cereus in fermented foods’, *Food microbiology*, 31(1), pp. 9–16.
3. Bigot, B. *et al.* (2011) ‘Control of Listeria monocytogenes growth in a ready-to-eat poultry product using a bacteriophage’, *Food microbiology*, 28(8), pp. 1448–1452.
4. Boyacioglu, O. *et al.* (2013) ‘Biocontrol of Escherichia coli O157: H7 on fresh-cut leafy greens’, *Bacteriophage*, 3(1), p. e24620.
5. Bueno, E. *et al.* (2012) ‘Phage inactivation of Staphylococcus aureus in fresh and hard-type cheeses’, *International journal of food microbiology*, 158(1), pp. 23–27.
6. Carter, C.D. *et al.* (2012) ‘Bacteriophage cocktail significantly reduces Escherichia coli O157’, *Bacteriophage*, 2(3), pp. 178–185. doi:10.4161/bact.22825.
7. Chibeu, A. *et al.* (2013) ‘Efficacy of bacteriophage LISTEX^TM^P100 combined with chemical antimicrobials in reducing Listeria monocytogenes in cooked turkey and roast beef’, *International Journal of Food Microbiology*, 167(2), pp. 208–214. doi:10.1016/j.ijfoodmicro.2013.08.018.
8. Endersen, L. *et al.* (2013) ‘Isolation and characterisation of six novel mycobacteriophages and investigation of their antimicrobial potential in milk’, *International Dairy Journal*, 28(1), pp. 8–14. doi:10.1016/j.idairyj.2012.07.010.
9. Ferguson, S. *et al.* (2013) ‘Lytic bacteriophages reduce Escherichia coli O157: H7 on fresh cut lettuce introduced through cross-contamination’, *Bacteriophage*, 3(1), p. e24323.
10. Figueiredo, A.C.L. and Almeida, R.C.C. (2017) ‘Antibacterial efficacy of nisin, bacteriophage P100 and sodium lactate against Listeria monocytogenes in ready-to-eat sliced pork ham’, *Brazilian journal of microbiology: [publication of the Brazilian Society for Microbiology]*, 48(4), pp. 724–729.
11. Galarce, N.E. *et al.* (2014) ‘Bacteriophage cocktail reduces Salmonella enterica serovar Enteritidis counts in raw and smoked salmon tissues’, *Revista Argentina de microbiologia*, 46(4), pp. 333–337.
12. Grant, A. ’quette *et al.* (2017) ‘Reduction of Salmonella in ground chicken using a bacteriophage’, *Poultry science*, 96(8), pp. 2845–2852.
13. Guenther, S. and Loessner, M.J. (2011) ‘Bacteriophage biocontrol of Listeria monocytogenes on soft ripened white mold and red-smear cheeses’, *Bacteriophage*, 1(2), pp. 94–100.
14. Guenther, S. *et al.* (2012) ‘Biocontrol of Salmonella Typhimurium in RTE foods with the virulent bacteriophage FO1-E2’, *International journal of food microbiology*, 154(1-2), pp. 66–72.
15. Hagens, S. and Loessner, M.J. (2010) ‘Bacteriophage for biocontrol of foodborne pathogens: calculations and considerations’, *Current pharmaceutical biotechnology*, 11(1), pp. 58–68.
16. Hagens, S., de Vegt, B. and Peterson, R. (2018) ‘Efficacy of a Commercial Phage Cocktail in Reducing Salmonella Contamination on Poultry Products- Laboratory Data and Industrial Trial Data’, *Meat and Muscle Biology*, 2, pp. 156–156. doi:10.22175/rmc2018.136.
17. Hong, Y. *et al.* (2016) ‘Treatment of *Salmonella*-Contaminated Eggs and Pork with a Broad-Spectrum, Single Bacteriophage: Assessment of Efficacy and Resistance Development’, *Foodborne Pathogens and Disease*, 13, pp. 679–688. doi:10.1089/fpd.2016.2172.
18. Hungaro, H.M. *et al.* (2013) ‘Use of bacteriophages to reduce Salmonella in chicken skin in comparison with chemical agents’, *Food Research International*, 52, pp. 75–81. doi:10.1016/j.foodres.2013.02.032.
19. Islam, M.S. *et al.* (2019) ‘Application of a Phage Cocktail for Control of Salmonella in Foods and Reducing Biofilms’, *Viruses*, 11(9), p. 841. doi:10.3390/v11090841.
20. Kang, H. W. *et al.* (2013) ‘wksl3, a New biocontrol agent for Salmonella enterica serovars enteritidis and typhimurium in foods: characterization, application, sequence analysis, and oral acute toxicity study’, *Applied and environmental microbiology*, 79(6), pp. 1956–1968.
21. Magnone, J.P. *et al.* (2013) ‘Additive approach for inactivation of Escherichia coli O157:H7, Salmonella, and Shigella spp. on contaminated fresh fruits and vegetables using bacteriophage cocktail and produce wash’, *Journal of food protection*, 76(8), pp. 1336–1341.
22. McLean, S.K., Dunn, L.A. and Palombo, E.A. (2013) ‘Phage inhibition of Escherichia coli in ultrahigh-temperature-treated and raw milk’, *Foodborne pathogens and disease*, 10(11), pp. 956–962.
23. Oliveira, M. *et al.* (2014) ‘Effectiveness of a bacteriophage in reducing Listeria monocytogenes on fresh-cut fruits and fruit juices’, *Food Microbiology*, 38, pp. 137–142. doi:10.1016/j.fm.2013.08.018.
24. Perera, M.N. *et al.* (2015) ‘Bacteriophage cocktail significantly reduces or eliminates Listeria monocytogenes contamination on lettuce, apples, cheese, smoked salmon and frozen foods’, *Food microbiology*, 52, pp. 42–48.
25. Shebs-Maurine, E.L. *et al.* (2020) ‘Application of MS bacteriophages on contaminated trimmings reduces Escherichia coli O157 and non-O157 in ground beef’, *Meat science*, 170, p. 108243.
26. Silva, E.N.G. *et al.* (2014) ‘Control of Listeria monocytogenes growth in soft cheeses by bacteriophage P100’, *Brazilian journal of microbiology: [publication of the Brazilian Society for Microbiology]*, 45(1), pp. 11–16.
27. Soffer, N. *et al.* (2016) ‘Bacteriophages safely reduce Salmonella contamination in pet food and raw pet food ingredients’, *Bacteriophage*, 6(3), p. e1220347.
28. Soffer, N. *et al.* (2017) ‘Bacteriophage preparation lytic for Shigella significantly reduces Shigella sonnei contamination in various foods’, *PloS one*, 12(3), p. e0175256.
29. Soni, K.A. and Nannapaneni, R. (2010) ‘Bacteriophage significantly reduces Listeria monocytogenes on raw salmon fillet tissue’, *Journal of food protection*, 73(1), pp. 32–38.
30. Soni, K.A., Nannapaneni, R. and Hagens, S. (2010) ‘Reduction of Listeria monocytogenes on the surface of fresh channel catfish fillets by bacteriophage Listex P100’, *Foodborne pathogens and disease*, 7(4), pp. 427–434.
31. Spricigo, D.A. *et al.* (2013) ‘Use of a bacteriophage cocktail to control Salmonella in food and the food industry’, *International Journal of Food Microbiology*, pp. 169–174. doi:10.1016/j.ijfoodmicro.2013.05.009.
32. Sukumaran, A.T. *et al.* (2015) ‘Reduction of Salmonella on chicken meat and chicken skin by combined or sequential application of lytic bacteriophage with chemical antimicrobials’, *International journal of food microbiology*, 207, pp. 8–15.
33. Sukumaran, A.T. *et al.* (2016) ‘Reduction of Salmonella on chicken breast fillets stored under aerobic or modified atmosphere packaging by the application of lytic bacteriophage preparation SalmoFreshTM’, *Poultry science*, 95(3), pp. 668–675.
34. Viazis, S. *et al.* (2011) ‘Reduction of Escherichia coli O157:H7 viability on leafy green vegetables by treatment with a bacteriophage mixture and trans-cinnamaldehyde’, *Food microbiology*, 28(1), pp. 149–157.
35. Vikram, A. *et al.* (2020) ‘Phage Biocontrol Improves Food Safety by Significantly Reducing the Level and Prevalence of Escherichia coli O157:H7 in Various Foods’, *Journal of Food Protection*, 83(4), pp. 668–676. doi:10.4315/0362-028x.jfp-19-433.
36. Ye, J. *et al.* (2010) ‘Control of Salmonella on Sprouting Mung Bean and Alfalfa Seeds by Using a Biocontrol Preparation Based on Antagonistic Bacteria and Lytic Bacteriophages’, *Journal of Food Protection*, 73(1), pp. 9–17. doi:10.4315/0362-028x-73.1.9.
37. Yeh, Y. *et al.* (2018) ‘Effect of ultraviolet light, organic acids, and bacteriophage on Salmonella populations in ground beef’, *Meat Science*, 139, pp. 44–48. doi:10.1016/j.meatsci.2018.01.007.
38. Zhang, X. *et al.* (2019) ‘SalmoFresh^TM^ effectiveness in controlling Salmonella on romaine lettuce, mung bean sprouts and seeds’, *International journal of food microbiology*, 305, p. 108250
